# Catalytic depolymerization of Kraft lignin to high yield alkylated-phenols over CoMo/SBA-15 catalyst in supercritical ethanol†

**DOI:** 10.1039/d3ra05018a

**Published:** 2023-10-13

**Authors:** Masud Rana, Shubho Ghosh, Theoneste Nshizirungu, Jeong-Hun Park

**Affiliations:** a Department of Environment and Energy Engineering, Chonnam National University Gwangju 61186 South Korea parkjeo1@jnu.ac.kr +82-62-530-1859 +82-62-530-1855

## Abstract

Lignin is generally considered to be a renewable and sustainable resource of aromatic chemicals. However, the depolymerization of Kraft lignin (KL) for the production of selective phenolic monomers presents a significant challenge due to its highly recalcitrant nature. Therefore, in this work, we investigated the effect of metal sites and acid active sites on Mo/SBA-15, Co/SBA-15 and CoMo/SBA-15 catalysts in supercritical ethanol for the depolymerization of KL to produce phenolic monomers. Ethanol was used as a hydrogen donor solvent instead of using external hydrogen. Results showed that the bimetallic CoMo/SBA-15 catalyst exhibited significantly higher catalytic activity compared to the monometallic, Co/SBA-15, Mo/SBA-15 or bare SBA-15. The highest phenolic monomers yield of 27.04 wt% was achieved at 290 °C for 4 h over CoMo/SBA-15 catalyst. The inter-unit linkages such as β-O-4′, β–β and α-O-4′ in lignin were considerably cleaved during the catalytic depolymerization in supercritical ethanol. Meanwhile, higher functionality of carbonyl compounds was present in the non-catalytic bio-oil, while more alkylated phenols were produced over CoMo/SBA-15 catalyst. The major phenolic monomers identified in the catalytic bio-oil were 4-ethylguaiacol (9.15 wt%), 4-methylguaiacol (6.80 wt%), and 4-propylguaiacol (2.85 wt%). These findings suggest that the metal sites and abundant acid active sites of CoMo/SBA-15 had a synergistic effect toward the degradation of different linkages of lignin and production of selective phenolic monomers in bio-oils.

## Abstract 2

Kraft lignin was subjected to CoMo/SBA-15 catalyst in supercritical ethanol for high yield of alkylated-phenolic monomers production.

## Graphical abstract

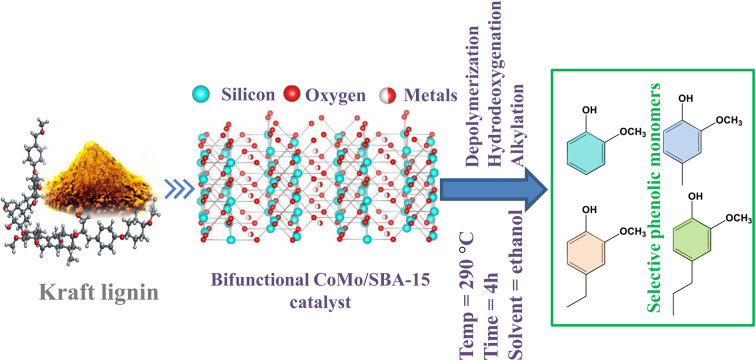

## 1. Introduction

Lignin is a major constituent of lignocellulosic biomass that accounts for 10–30% of its weight and contributes to approximately 40% of its energy content.^1,2^ Among different lignins, Kraft lignin (KL) is generated as an industrial byproduct in pulp mills, with an estimated annual production exceeding 40–50 million tons worldwide.^3,4^ Despite the abundant availability of KL compared to fossil fuels, a significant portion of it remains underutilized, often being used as low-grade fuel in recovery boilers, leading to severe environmental concerns. Notably, KL holds promise as a potential source of high-grade chemicals if it can be converted into smaller units. However, the main challenge associated with the valorization of KL is its highly variable structure. The chemical compositions of different available KLs depend on their botanical origin, extraction techniques and delignification methods. Therefore, to address the significant heterogeneity of KL and enhance the economic viability of Kraft mills, significant efforts have been made to depolymerize KL into various value-added chemicals.

To date, several approaches have been developed for the depolymerization of lignin including fast pyrolysis,^5^ oxidation,^6–8^ solvothermal,^9,10^ and hydrothermal liquefaction.^11,12^ Of these approaches, solvothermal has received significant attention as a technique for lignin degradation, which involves the use of external hydrogen or a hydrogen donor solvent with or without the presence of a metal catalyst. Supercritical alcohols such as methanol,^13,14^ ethanol^15,16^ or isopropanol^17,18^ are being widely used for lignin depolymerization due to their favorable hydrogen donor behavior, excellent solubilization capabilities and efficient heat transfer. Taking into account the positive role of supercritical alcohols, Warner *et al.*^14^ studied the depolymerization of organosolv lignin (source: candlenut nutshells) in supercritical methanol using Cu- and La-doped hydrotalcite-like catalysts and achieved a complete lignin conversion into bio-oil at 310 °C for 6 h. Compared to methanol or isopropanol; ethanol is proven to be an effective solvent for the production of high yield of phenolic monomers and the suppression of repolymerization during the lignin depolymerization reaction. For example, Feng *et al.*^19^ studied KL (source: extracted from the wheat straw biomass) using NiAl/MCM-41 catalyst at different temperatures (250–310 °C), times (30–90 min), and solvents (water, ethanol and methanol). They achieved a bio-oil yield of 56.2 wt% and reported that ethanol is more effective in preventing repolymerization than other solvents. In our previous work,^1^ we investigated concentrated acid hydrolysis lignin (source: black liquor) liquefaction in water and organic solvents (such as methanol, ethanol, and isopropanol), and found that ethanol gave less char formation and high monomers yield as compared to those obtained with other solvents. Based on the findings presented above, ethanol can be considered as one of the best performing solvent for lignin conversion to phenolic monomers under supercritical conditions.

In recent years, there has been increasing interest in Co- and Mo-based catalysts supported on various carriers for the hydrogenolysis of lignin and the enhancement of bio-oil quality.^9,20–22^ These catalysts have attracted attention due to their crystalline structures and ability to exist in multiple valence states. For example, Wang *et al.*^22^ evaluated the effect of Mo/sepiolite catalyst on the depolymerization of KL (Indulin AT) in supercritical ethanol and it was concluded that the presence of Lewis active sites and Mo species (Mo^6+^ and Mo^5+^) facilitated the deoxygenation and alkylation reactions during lignin depolymerization, resulting in the production of phenolic monomers. Zhang *et al.*^23^ compared the role of Co/MCM-41, Co/SBA-15 and Co/FDU-1 catalyst on the hydrodeoxygenation of vanillin (a lignin-derived model compound) and found that Co/SBA-15 had the best catalytic activity. Yang *et al.*^24^ reported that Co/SBA-15 catalyst could selectively hydrogenolysis the C–O bond in the lignin and can acts as a hydrodeoxygenation catalyst of lignin-derived bio-oils, due to its high efficiency in the removal of oxygen by a direct hydrogenolysis route. Besides excellent catalytic activities of monometallic catalyst, bimetallic catalysts offer the possibility to accelerate the lignin hydrogenolysis process due to their combined effect. Considering the beneficial effect of bimetallic catalyst, Pu *et al.*^25^ used CoMoS/Al_2_O_3_ catalyst for the depolymerization of protobind 1000 lignin (source: wheat straw soda lignin) in a semi-continuous reactor at 350 °C for 13 h and they obtained a 62.5 wt% yield of organic products. The same group^26^ also developed a kinetic model for the hydroconversion of protobind 1000 lignin over CoMoS/Al_2_O_3_ catalyst in a semi-batch reactor. Hao *et al.*^27^ studied the catalytic performance of the Co–Mo–S/AC, Co–Mo–S/γ-Al_2_O_3_, and Co–Mo–S/ZrO_2_ catalysts on the depolymerization of dealkaline lignin (source: needle-leaved trees and broad-leaved trees) and obtained 95.76 wt% liquid products at 340 °C for 2.5 h over Co–Mo–S/ZrO_2_ catalyst. Jongerius *et al.*^28^ developed a two-steps process for the depolymerization of various lignins, including KL (source: Indulin AT) to produce monomeric compounds over Pt/γ-Al_2_O_3_ catalyst in water/ethanol system and the achieved bio-oil was further deoxygenated with CoMo/Al_2_O_3_ catalyst. However, a common challenge faced by researchers in the field of bio-refineries is the low surface area and potential catalyst deactivation due to the clogging of support material. Therefore, the choice of a suitable metal carrier or support is also crucial in determining the catalytic activity of catalysts.

Currently, mesoporous silicate materials such as MCM-41, Al/MCM-41 and SBA-15 have gained attention as supports/carriers due to their high surface area, excellent thermal stability and adjustable pore sizes. For example, Kim *et al.*^29^ evaluated the effect of supercritical ethanol over Ru–Ni/SBA-15 catalyst for the depolymerization of soda lignin (source: wheat straw and sarkanda grass) into phenol-rich bio-oil. They obtained 77.5 wt% yield of bio-oil at 350 °C for 40 min. Lu *et al.*^30^ investigated lignin (source: Chinese fir sawdust) depolymerization in isopropanol/formic acid solvent mixture over NiMo/Al-MCM-41 catalyst, and attained a bio-oil yield of 61.6 wt%. In another study, Klamrassamee *et al.*^31^ compared the effect of MCM-41, SBA-15, ZrO_2_-MCM-41 and ZrO_2_-SBA-15 on the depolymerization of organosolv lignin (source: woody eucalyptus) at temperatures of 200–350 °C and time of 1 h. They found that SBA-15 gave the less char yield as compared to that obtained with the MCM-41, ZrO_2_-MCM-41 and ZrO_2_-SBA-15.

KL is marketed in two different forms (which have different psychochemical properties, Fig. S1†) under the product numbers 471003 and 370 959. However, at the present time, the KL with product number 471003 has been extensively investigated for the production of valuable aromatic chemicals. For instance, Ma *et al.*^32^ utilized a MoC/AC catalyst for the depolymerization of KL (Sigma-Aldrich, product no. 471003) in supercritical ethanol at 280 °C for 6 h, achieving high yields (61.3 wt%) of aromatic compounds without char formation. Biswas *et al.*^33^ prepared a Co/CaO catalyst for the depolymerization of KL (Sigma-Aldrich, product no. 471003) in water, ethanol and methanol solvents. They reported a maximum bio-oil yield of 60.2 wt% in methanol using 10 wt% Co/CaO, highlighting the excellent catalytic capability of Co for the hydrodeoxygenation of depolymerized products of lignin. Chen *et al.*^34^ examined KL (Sigma-Aldrich, product no. 471003) depolymerization into small-molecular liquid products in supercritical ethanol over Mo_2_N/Al_2_O_3_ catalyst. Dou *et al.*^35^ prepared a ZnCoOx catalyst for the KL (Sigma-Aldrich, product no. 471003) depolymerization in a mixed solvent of methanol and 1,4-dioxane. They achieved a 72% yield of liquid products at 300 °C for 6 h and reported the essential role of Co to the cleavage of ether linkages in lignin *via* hydrogenolysis. Although, KL-471003 has been widely utilized for the aromatic chemicals, so far, research has not been reported on the depolymerization of KL (product no. 370959) into aromatic chemicals. In a recent study, Ghoreishi *et al.*^36^ investigated depolymerization of lignin extracted from various sources such as Norway Spruce-BL, Eucalyptus and Alkali lignin, in water–ethanol–formic acid and showed that the yield and composition of liquid products obtained after each lignin depolymerization are quite different. Therefore, extensive research is required for each lignin species, as lignin structure and its depolymerization products greatly depend on the plant types and the lignin isolation methods.

Inspired by the intriguing catalytic properties of Co- and Mo-based catalysts and the SBA-15 support, this study focused the effects of mono and bifunctional Mo/SBA-15, Co/SBA-15 and CoMo/SBA-15 catalysts on the depolymerization of commercially available KL (product no. 370959) to produce phenolic monomers in supercritical ethanol. Different reaction parameters such as temperature and time were investigated to determine the optimal conditions. The prepared catalysts were characterized *via* BET, XPS, XRD and HR-TEM analyses, while the produced bio-oils were analyzed by GC-MS, FT-IR, ^13^C NMR, 2D HQSC NMR, and elemental analyses.

## 2. Experimental section

### 2.1 Chemicals and materials

The Kraft lignin (source: pine wood, CAS no. 8068-05-1, product number: 370959) used in this study was purchased from Sigma-Aldrich. All chemicals and reagent including ethanol (≥98%), ethyl acetate (CH_3_COOC_2_H_5_), ammonium molybdate ((NH_4_)_6_Mo_7_O_24_·4H_2_O), cobalt nitrate (Co(NO_3_)_2_·6H_2_O), pluronic 123, and tetraethyl orthosilicate (TEOS) were also purchased from Sigma-Aldrich (USA, ≥99.5%). All chemicals were used as received without further purification.

### 2.2 Preparation of catalyst

The catalyst support porous SBA-15 material was synthesized according to our previous work.^37^ Herein, 10 g of P123 was added to 120 mL of 2 M aqueous HCl solution and 30 mL of deionized (DI) water and stirred at room temperature for 15 min. Subsequently, 20 g of TEOS was added to the solution and the mixture was heated at 40 °C with a moderate stirring speed of 300 rpm for 2 days. The resulting solution was then transferred to a Teflon-lined autoclave and heated at 100 °C for 2 days. Finally, the solid product was filtered, washed multiple times with DI water, dried in air at 105 °C overnight and calcined in a muffle furnace at 550 °C for 4 h.

For the preparation of CoMo/SBA-15 catalyst, the porous SBA-15 was impregnated with aqueous solutions of (NH_4_)_6_Mo_7_O_24_·4H_2_O (0.39 mmol) and Co(NO_3_)_2_·6H_2_O (1.68 mmol). Cobalt and molybdenum loadings in the SBA-15 were fixed at 5 wt%, respectively, (total metal loading = 10 wt% and Co : Mo ratio = 1 : 1). The impregnated precursors were then stirred at 60 °C for 2 h followed by prolonged drying at 100 °C for 12 h. Finally, the resulting solid product was calcined at 550 °C (10 °C min^−1^) for 4 h under static air conditions. For comparison, monometallic Co and Mo catalysts supported on SBA-15 (denoted as Co/SBA-15 and Mo/SBA-15, respectively) were also prepared by an incipient wetness impregnation method according to the similar method described above.

### 2.3 Lignin depolymerization

All experiments were performed in a 150 mL batch reactor (Hastelloy-C-276 HR-8300) equipped with a reactor controller (Parr 4848). Initially, 3.0 g of KL and 90 mL of ethanol with or without SBA-15 supported metal catalysts were loaded into a stainless-steel reactor. The reactor was then sealed and purged with N_2_ to expel the air inside. The reactor was heated to the intended reaction temperature at a rate of 10 °C min^−1^ by an electric furnace, and the reactor was maintained at this temperature for the specified reaction time. When the preset reaction conditions were completed, the reactor was cooled to room temperature in an ice-water bath. Finally, the reactor was vented and opened to collect the desired reaction products.

**Fig. 1:**
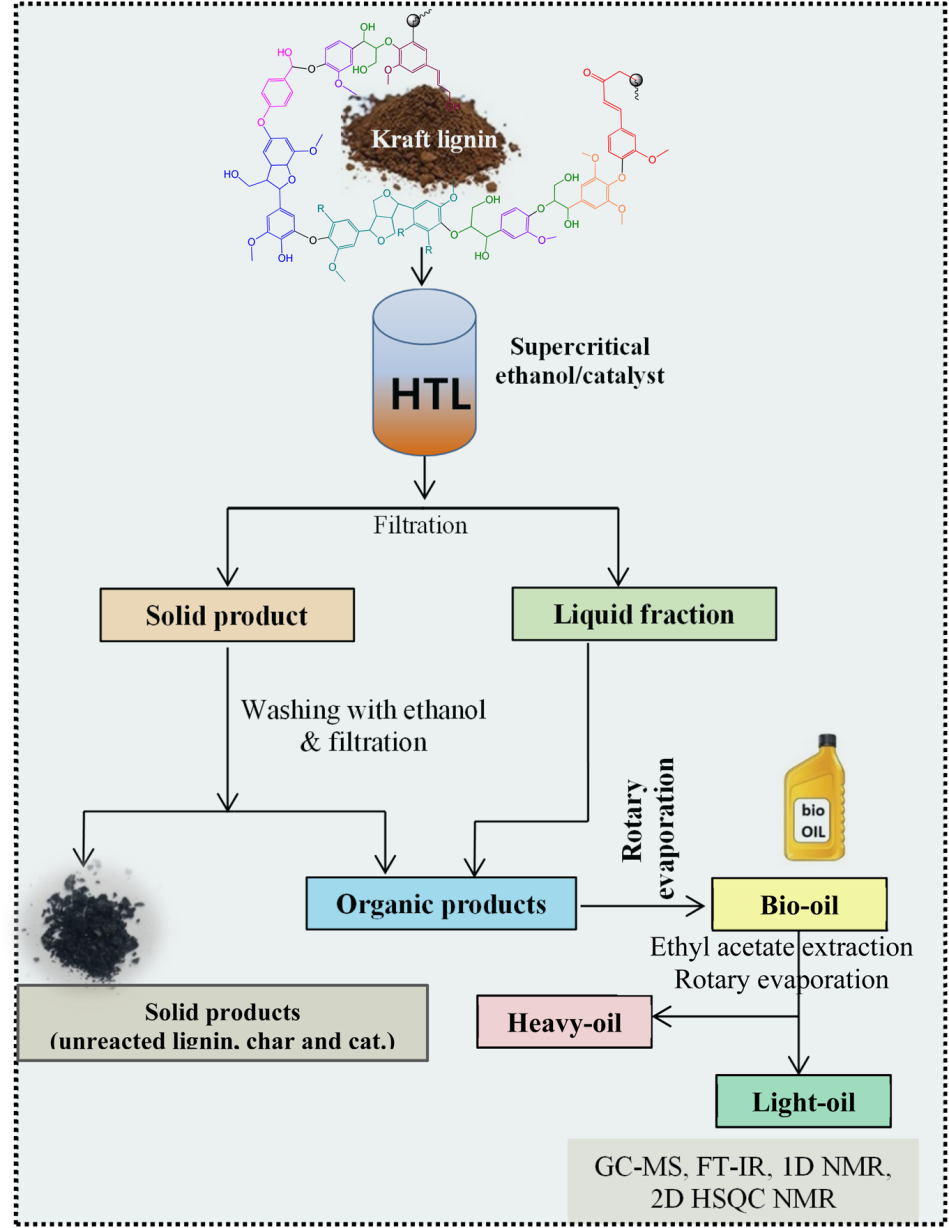
Reaction product mixture processing sequence and analysis.

Fig. 1 shows the detailed product separation techniques. The reaction mixture was first filtered to separate the liquid fraction from the solid product. The solid product was washed with ethanol and then dried in an oven at 110 °C and weighed. The solid residue/product was composed of unreacted lignin, char and catalyst. Meanwhile, the liquid fraction was evaporated in a rotary evaporator at a reduced pressure and 60 °C to remove the ethanol solvent and to obtain bio-oil, a brown liquid. The bio-oil thus obtained was weighed and collected for further analysis. Lignin-derived bio-oil typically consists of a volatile fraction (monomers) and a non-volatile fraction (oligomers). To extract the volatile fraction, 20 mL of ethyl acetate solution was added to the bio-oil, followed by centrifugation and filtration. The resulting ethyl acetate soluble fraction was subjected to rotary evaporation to obtain the volatile fraction, referred to as light-oil. While the ethyl acetate insoluble products was defined as heavy-oil. The yields of various products were calculated using the following eqn (1–5):1
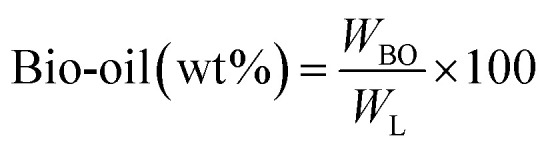
2
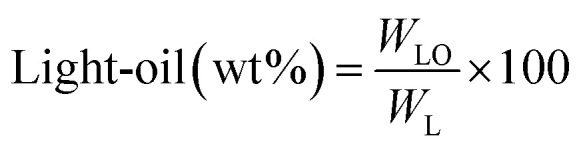
3
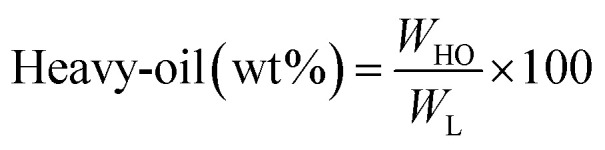
4
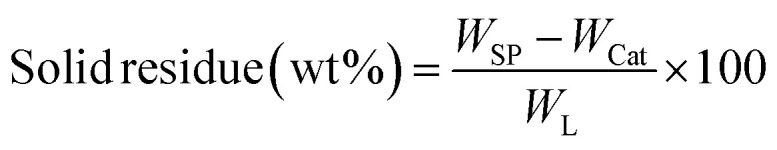

5 Gas (wt%) = 100 − (bio-oil + solid product)

where, *W*_BO_ = weight of the bio-oil, *W*_L_ = weight of the feed lignin, *W*_SP_ = weight of the solid product, *W*_Cat_ = weight of the feed catalyst, *W*_LO_ = weight of the light-oil and *W*_HO_ = weight of the heavy-oil. Each experiment was repeated three times and the average value is presented in the result.

### 2.4 Characterization of catalyst

The textural properties of the synthesized catalysts, such as surface area and pore size distribution were determined by conducting N_2_ adsorption–desorption isotherms measurements at 77.35 K using a BELCAT-A analyzer. The surface area was determined using the Brunauer–Emmett–Teller (BET) method, while the pore size distribution was calculated using the Barrett–Joyner–Halenda (BJH) method, based on the adsorption branches of the nitrogen adsorption–desorption isotherm. Prior to testing, all samples were degassed in a vacuum (10^−6^ Torr) at 200 °C for 12 h. The total pore volume was measured at a relative pressure (*P*/*P*_o_) of 0.990.

The powder X-ray diffraction (XRD) patterns of the synthesized catalysts were obtained using a 3D high resolution X-ray diffractometer (model: EMPYREAN) with a PIXcel^3D^, PREFIX INTERFACE Xenon proportional detector and Cu Kα radiation (*λ* = 1.5406 Å) in the 2*θ* ranges of 0.5–5° (for low angles) and 5–90° (for wide angles). The XRD data were qualitative analyzed using High Score Plus 4.1 software (analyzed with the latest ICDD Card Database). Transmission electron microscopy (TEM) micrographs were captured with a JEOL JEM-2100F electron microscope operated at an acceleration voltage of 200 kV. The elemental compositions of the synthesized catalysts were measured with an EDS detector (ATW2, 133 eV, 10 mm^2^). An X-ray photoelectron spectroscopy (XPS) analysis of the as-synthesized catalysts was performed using a PerkinElmer PHI-1600 spectrometer with monochromatic Mg Kα radiation.

Temperature-programmed desorption (TPD) experiments were conducted using NH_3_-TPD to determine the acidity of the samples. The catalysts were initially heated to 500 °C and then cooled down to 100 °C under a He atmosphere. Subsequently, 5% NH_3_/He (20 mL min^−1^) was adsorbed at 100 °C for 30 min, followed by He purging at the same temperature for 1 h. The desorption of ammonia was monitored in the temperature range of 100–900 °C at a heating rate of 10 °C min^−1^. Additionally, ammonia desorption in the samples was analyzed using a thermal conductivity detector (TCD). Inductively coupled plasma-optical emission spectrometry (ICP-OES) was carried out to determine the metals content in the prepared catalyst.

### 2.5 Product analyses

The qualitative and quantitative analyses of bio-oils were conducted using an Agilent 6890 gas chromatograph-mass spectrometer (GC-MS) equipped with an HP-5MS capillary column (30 m × 0.25 μm × 0.25 mm). High purity helium (He) was used as the carrier gas (at a flow rate of 1 mL min^−1^). The column temperature was initially set to 40 °C for 2 min and then increased to 170 °C at a heating rate of 10 °C min^−1^, where it was held for 5 min. Finally, the temperature was further increased to 300 °C at a heating rate of 10 °C min^−1^ and maintained for an additional 2 min. The individual components in the bio-oils were identified by comparing their GC-MS spectra and retention times with those of authentic compounds available in the National Institute of Standards and Technology (NIST) mass spectral library. For quantification of the yield of selected phenolic compounds (phenol, guaiacol, 4-methylguaiacol, 4-ethylguaiacol, 4-propylguaiacol, eugenol, vanillin, acetovanillone, isoeugenol, and homovanillic acid), an external calibration method was used. The yield was calculated as follows:

6 Yield of monomer (wt%) = the weight of monomer/the weight of lignin × 100

The NMR analyses (^1^H and ^13^C) of raw KL and lignin oils were carried out at 300.9 K using the AVANCE III HD (400.0 MHz) spectrometer manufactured by Bruker (Germany). DMSO-*d*_6_ was used as the solvent. The NMR tubes had a diameter of 5 mm, and the pulse delay was set to 1 s with a spectral width of 24 038.5 Hz. HSQC NMR analyses of KL and the ESP fractions were performed using the same NMR analyzer mentioned above. Fourier-transform infrared spectroscopy (FT-IR) analyses were carried out using a 400 FT-IR spectrometer (PerkinElmer, USA). The spectra were logged at a resolution of 1 cm^−1^ within the wavelength range of 500–4000 cm^−1.^

The elemental analyses of the feed KL, heavy-oil and light-oil fractions of bio-oils were carried out a Vario MACRO cube analyzer (EL III, Germany). The elemental components, such as C, H, and N of lignin and lignin-derived bio-oils, were analyzed by the elemental analyzer, while the oxygen (O) content was calculated based on the difference. The high heating values (HHV) or calorific values of KL and lignin oils were calculated according to DIN 51900 standard (eqn (7)):

7 HHV (MJ kg^−1^) = [(34 × C) + (124.3 × H) + (6.3 × N) + (19.3 × S) − (9.8 × O)]/100

## 3. Results and discussion

### 3.1 Characterization of prepared catalysts

**Fig. 2:**
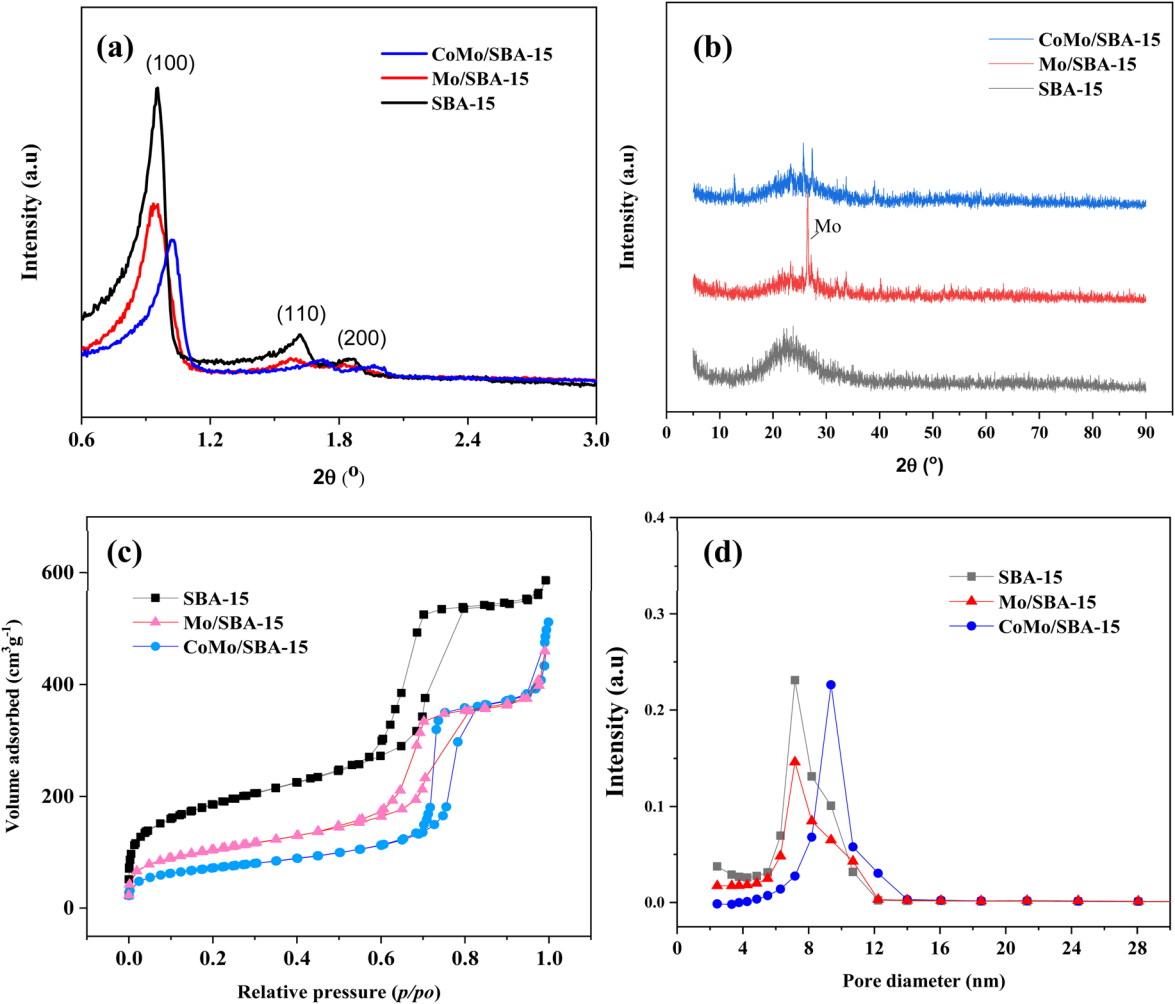
(a) Small and (b) wide-angle XRD patterns of SBA-15, Mo/SBA-15 and CoMo/SBA-15 catalysts, (c) N_2_ adsorption–desorption isotherms, and (d) pore size distributions of SBA-15, Mo/SBA-15 and CoMo/SBA-15 catalysts.

Fig. 2a and b show the small and wide-angle XRD patterns of the prepared catalysts SBA-15, Mo/SBA-15 and CoMo/SBA-15. The small-angle XRD pattern of the synthesized SBA-15 material exhibited distinct diffraction peaks at 2*θ* = 0.95°, 1.62° and 1.87°, which correspond to the (100), (110) and (200) planes, respectively, indicating a typical hexagonal mesoporous structure.^38^ When Mo or Co metal was deposited onto the SBA-15 support material, the d_100_, d_110_ and d_200_ peaks were still observed in the XRD patterns of Mo/SBA-15 and CoMo/SBA-15, indicating that the impregnation of Mo and Co did not disrupt the well-ordered porous structure of SBA-15. In the wide-angle XRD patterns (Fig. 2b), the SBA-15 material showed a broad diffraction peak at around 22°. The diffraction peaks observed at 2*θ* = 26.6°, 33.72° and 40.16° in the Mo/SBA-15 material confirm the presence of Mo particles on the SBA-15 material. However, the CoMo/SBA-15 material did not exhibit a diffraction pattern for Co, which could be attributed to the formation of small size Co nanoparticles. Moreover, Co particles could be well-dispersed over SBA-15 material and was not detected by XRD. A similar phenomenon was also observed in the Yang *et al.*^24^ work, where the Co/SBA-15 and Ni/SBA-15 catalysts were synthesized for the hydrodeoxygenation of lignin-derived bio-oil and it was found that the diffraction peak of Co was absent in the XRD pattern of Co/SBA-15 catalyst. They concluded that Co was very well dispersed over SBA-15 material and it was hardly detected by XRD. In another study, Hao *et al.*^9^ prepared Co–Mo–S/AC and Co–Mo–S/ZrO_2_ catalysts for the depolymerization of dealkaline lignin. In the XRD pattern of CoMo–S/AC catalyst, they also did not found the peak of Co–Mo–S and reported that the loaded metals were distributed evenly and well over AC support. In the present study, the XPS analysis was further carried out to confirm the deposition of Mo and Co, and the successful synthesis of the SBA-15 material.

**Fig. 3:**
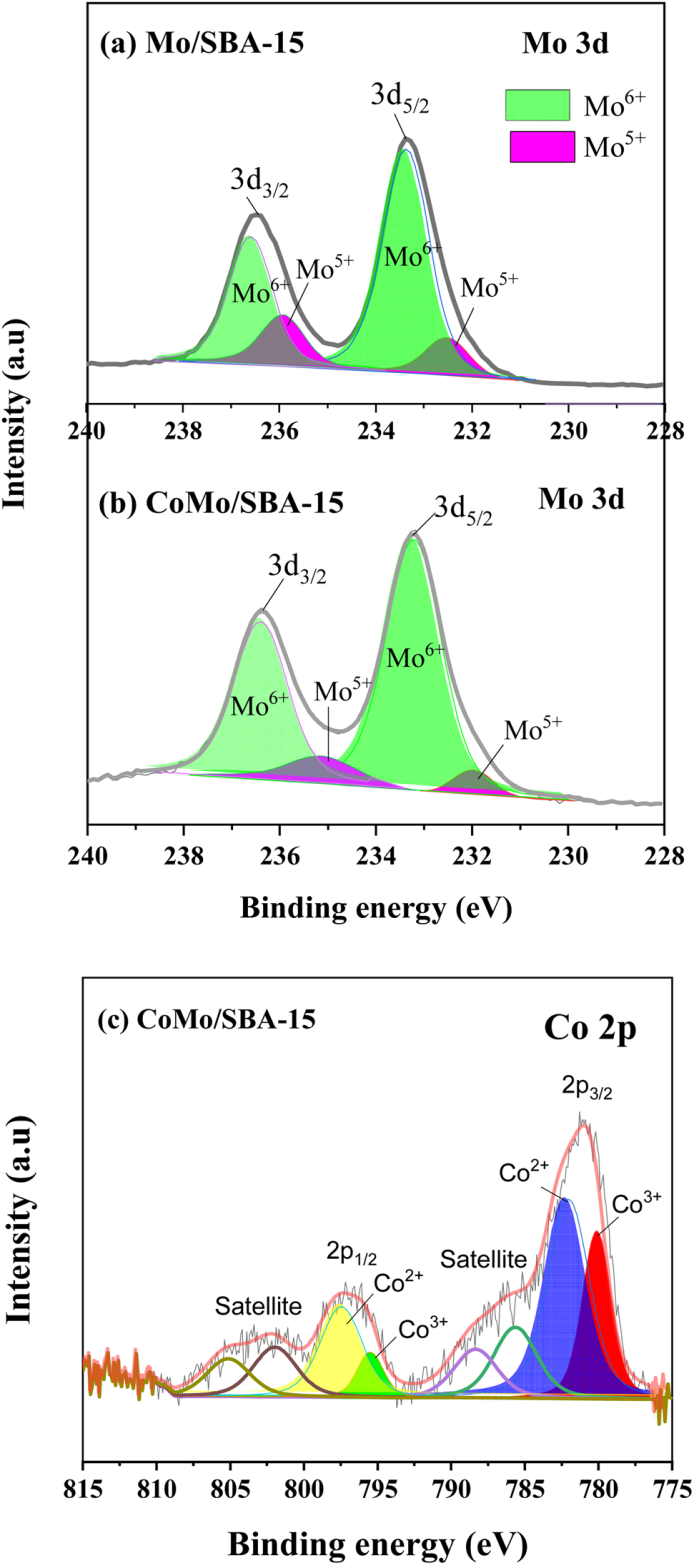
Core level spectra of Mo 3d in (a) Mo/SBA-15 and (b) CoMo/SBA-15, (c) core level spectrum of Co 2p in CoMo/SBA-15.

Fig. 3 shows the Mo 3d and Co 2p core level spectra in the Mo/SBA-15 and CoMo/SBA-15 materials, while Fig. S2 in the ESI† presents the Si 2p and O 1s binding energy (BE) peaks in the SBA-15 sample. The Si 2p and O 1s BE peaks identified at 103.99 eV and 533.11 eV, respectively, correspond to the O–Si–O and O–Si bonds in the SBA-15 phase.^37^ From Fig. 3a–c, it is evident that both the Mo/SBA-15 and CoMo/SBA-15 materials exhibited distinct Mo 3d and Co 2p BE peaks, confirming the deposition of Mo and Co onto the SBA-15 material. As for Mo/SBA-15 (Fig. 3a), the Mo 3d BE peaks were observed at 231.98 and 235.16 eV, as well as at 233.21 and 236.39 eV, correspond to the Mo(v) 3d_5/2_ and Mo(vi) 3d_3/2_ oxidation states, respectively. Similarly, in the CoMo/SBA-15 (Fig. 3b), the Mo 3d BE peaks were observed at 232.52 and 235.93 eV, as well as at 233.36 and 236.59 eV, assign to Mo(v) 3d_5/2_ and Mo(vi) 3d_3/2_ oxidation states, respectively.^22^ The CoMo/SBA-15 material also shows the BE peak of Co 2p at (780.46 and 795.48 eV) for Co 2p_3/2_ and (782.08 and 797.58 eV) for Co 2p_1/2_, which are attributed to the Co(iii) and Co(ii) oxidation states of the Co_3_O_4_ and CoO phases^39,40^ within the CoMo/SBA-15 material (Fig. 3c).

The nitrogen adsorption–desorption isotherms and pore size distributions of the SBA-15, Mo/SBA-15 and CoMo/SBA-15 catalysts are shown in Fig. 2c and d. The SBA-15 material exhibited a type-IV adsorption–desorption isotherm with H1-type hysteresis loop. After loading the metal, the hysteresis loop of SBA-15 remained intact in the Mo/SBA-15 and CoMo/SBA-15 catalysts, indicating that the Mo/SBA-15 and CoMo/SBA-15 catalysts maintained the inherent pore characteristics of SBA-15.

The physicochemical properties of the SBA-15, Co/SBA-15, Mo/SBA-15 and CoMo/SBA-15 catalysts are summarized in Table 1. The unsupported SBA-15 material has a specific surface area of 658.51 m^2^ g^−1^, a total pore volume of 0.90 cm^3^ g^−1^, and an average pore diameter of 5.50 nm. When Mo or Co was incorporated onto the SBA-15 support, the specific surface area and total pore volume of SBA-15 were found to have decreased to 369.93 m^2^ g^−1^ and 0.70 cm^3^ g^−1^ in the Mo/SBA-15 material, 387.38 m^2^ g^−1^ and 0.77 cm^3^ g^−1^ in the Co/SBA-15, and 255.53 m^2^ g^−1^ and 0.67 cm^3^ g^−1^ in the CoMo/SBA-15 material. These results could be explained by the potential clogging of the SBA-15 mesopore channels caused by the loading of Mo and Co metals. Metal loadings of the prepared catalysts determined by ICP-OES analyses are listed in Table S1.†

**Table 1:** Physiochemical properties of SBA-15, Co/SBA-15, Mo/SBA-15 and CoMo/SBA-15 catalysts

Catalyst	*S*_BET_ (m^2^ g^−1^)	Total pore volume (cm^3^ g^−1^)	Mean pore diameter (nm)	NH_3_-TPD (mmol g^−1^)		
				Weak acid site	Strong acid site	Total acidity
SBA-15	658.51	0.90	5.50	0.05	0.64	0.69
Mo/SBA-15	369.93	0.70	7.67	0.48	0.63	1.11
Co/SBA-15	387.38	0.77	6.21	0.37	0.55	0.92
CoMo/SBA-15	255.53	0.67	10.60	1.10	0.33	1.43

To measure the strength of acid sites in SBA-15, Mo/SBA-15 and CoMo/SBA-15 catalysts, NH_3_-TPD analysis was conducted and the results are presented in Fig. S3† and Table 1. The parent SBA-15 support showed two broad desorption peaks in the temperature range of 160–300 °C (indicating weak acid sites) and 650–830 °C (indicating strong acid sites) with values of 0.05 and 0.64 mmol g^−1^, respectively. Upon the incorporation of Mo and Co into the SBA-15 material, a decrease in the peak intensity of the strong acid sites and a significant increase in the peak intensity of weak acid sites were observed. Previous research has reported that metal ions loaded onto an acidic support can act as Lewis acid sites,^41^ thus, the peak observed at 160–300 °C can be attributed to the presence of Lewis acid sites. Among the prepared catalysts, CoMo/SBA-15 catalyst exhibited the highest total acidity (*T* = 1.14 mmol g; sum of 1.10 mmol g^−1^ weak acid and 0.33 mmol g^−1^ strong acid), which was followed by the Mo/SBA-15 (*T* = 1.11 mmol g; sum of 0.48 mmol g^−1^ weak acid and 0.63 mmol g^−1^ strong acid) catalyst (Table 1).

**Fig. 4:**
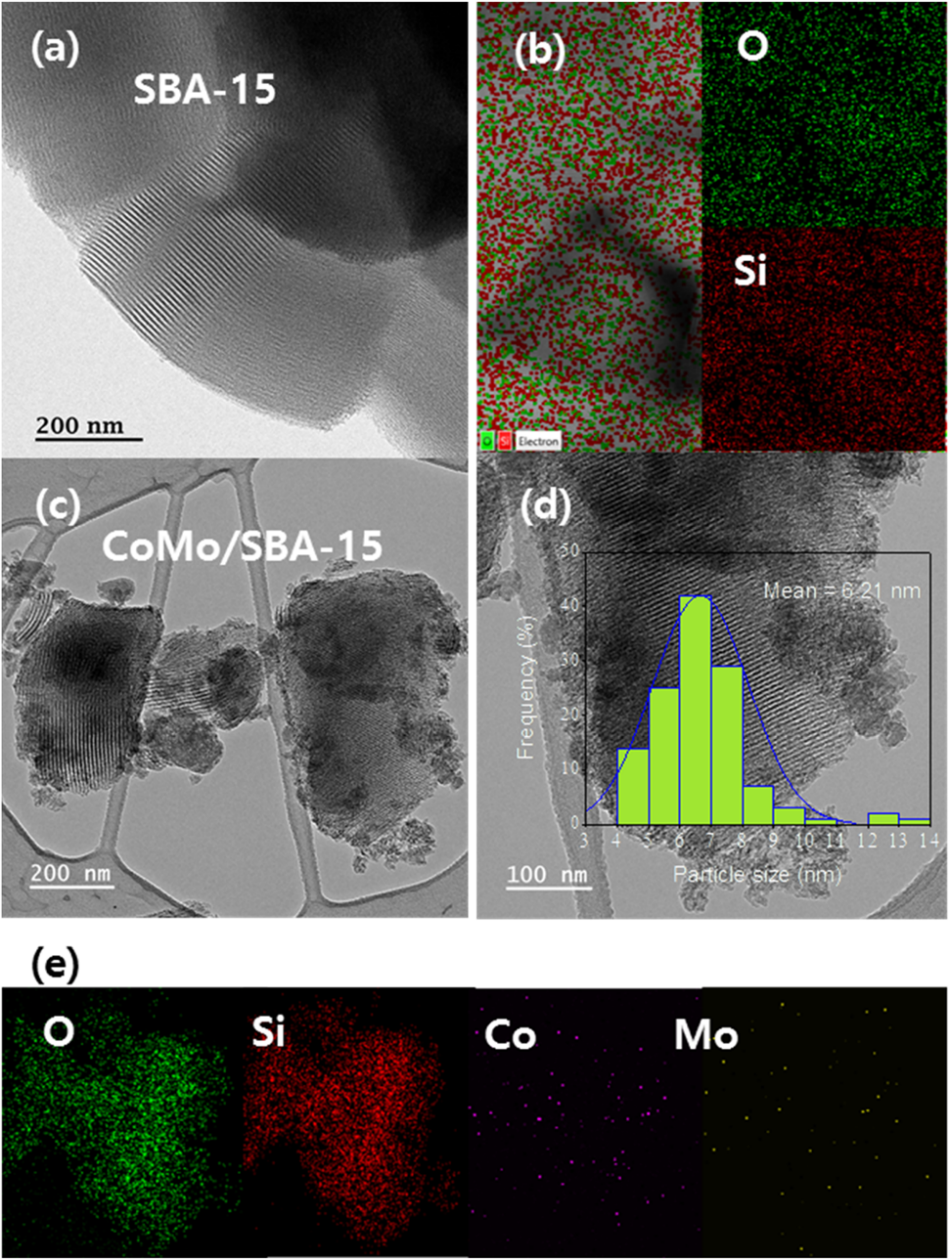
HR-TEM images and element mapping of (a and b) SBA-15 and (c–e) CoMo/SBA-15 catalyst.

TEM and EDS analyses were carried out to investigate the surface modifications that occur before and after the incorporation of metals into the SBA-15 material. Fig. 4 displays the HR-TEM images and EDS element mapping of the SBA-15 and CoMo/SBA-15 materials. The well-ordered pore channels are clearly discernible in the HR-TEM image of the synthesized SBA-15 material, which is in accordance with the nitrogen adsorption–desorption isotherms and low angle XRD patterns. In the case of the CoMo/SBA-15 material, the dark zones over and outside of the mesoporous structure indicating the deposition of metal particles on the surface of SBA-15. Moreover, the TEM image show that the average particle sizes of the loaded metals on the bulk SBA-15 support was 6.2 nm. While the EDS element mapping results confirmed the presence of Mo and Co particles in the CoMo/SBA-15 material. Furthermore, the EDS analysis of CoMo/SBA-15 (Fig. 4e) indicates that the Co and Mo particles are well dispersed on the SBA-15 material.

### 3.2 Effect of reaction temperature and time

**Fig. 5:**
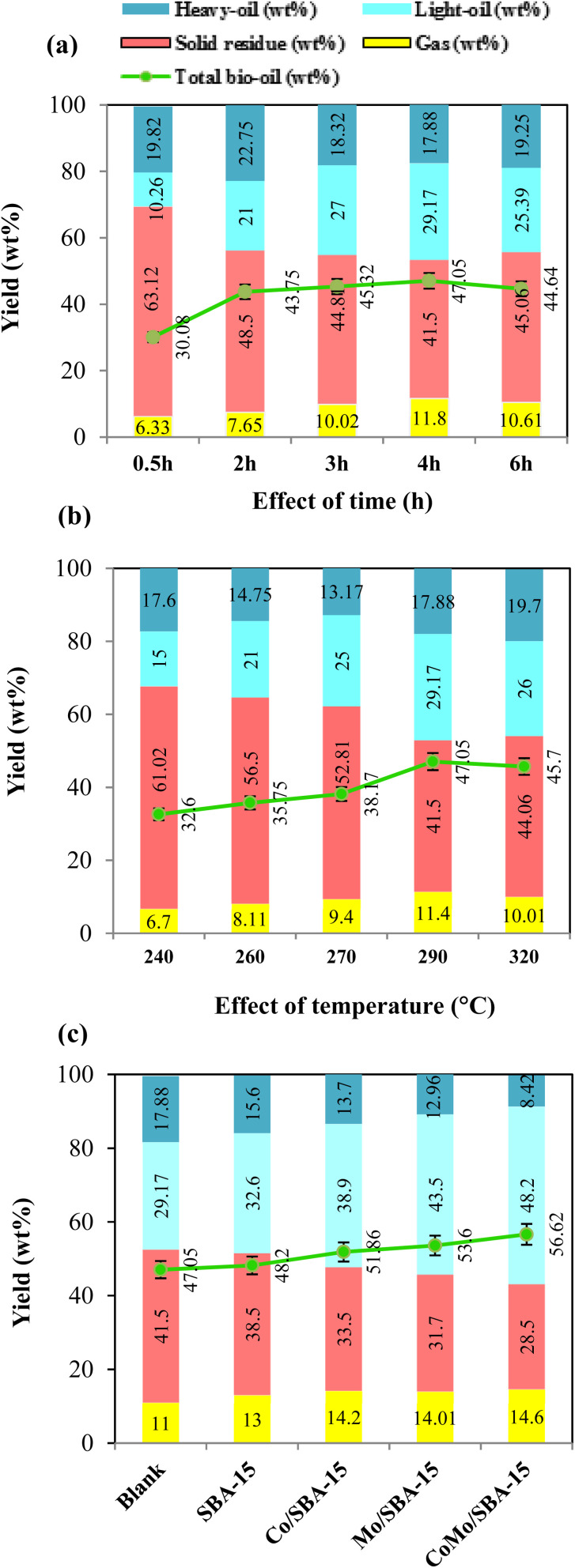
Effect of (a) time, (b) temperature, and (c) catalysts, on the yield of lignin depolymerized products. Conditions: (a) 3.0 g lignin, 290 °C and 90 mL ethanol; (b) 3.0 g lignin, 4 h and 90 mL ethanol; (c) 3.0 g lignin, 290 °C, 4 h and 90 mL ethanol.

The effect of reaction time on the yield of depolymerized lignin products was performed in ethanol by varying the time from 0.5 h to 6 h at 290 °C without catalyst, and the results are illustrated in (Fig. 5a and Table S2†). As observed, at the initial reaction time of 0.5 h, the yield of bio-oil was 30.08 wt% which included 10.26 wt% light-oil, indicating that lignin was partially depolymerized at shorter reaction time. With increasing the time from 0.5 h to 4 h, the yield of total bio-oil and light-oil improved to 47.05 and 29.17 wt%, respectively, while the solid residue yield decreased from 63.12 to 41.50 wt%. As the reaction time increased further from 4 h to 6 h, the yield of solid residue was found to be increased to 45.06 wt% while the light-oil decreased to 25.39 wt%. These results indicate that longer reaction time (more than 4 h) could favor the repolymerization of reactive monomers, resulting in high yield of char formation. The FT-IR results of the solid residues obtained at different reaction times (Fig. S4a of the ESI†) also support well the above statement. As depicted in Fig. S4a,† the FT-IR spectrum of the solid residue obtained at 0.5 h showed slight differences with the KL, indicating that the KL depolymerization was insignificant at shorter reaction time. As time grew up, the strength of hydroxyl group (3658–3028 cm^−1^), aromatic rings (1606 and 1128 cm^−1^) and C–O group (1037 cm^−1^) began to weaken obviously. The peaks of the aforementioned groups were less intense in the solid residue obtained at 4 h reaction time. However, as the time further increased to 6 h, the peak intensities of hydroxyl group and aromatic rings were found to be increased again, which gave a good indication for the occurrence of repolymerization at a longer reaction time (>4 h). Huang *et al.*^42^ and Roberts *et al.*^43^ also reported that phenolic hydroxyl groups are the main actors in repolymerization and char formation. In a previous work, Bartolomei *et al.*^44^ investigated the depolymerization of lignin in ethanol under the reaction conditions of 250 °C for (1–4 h) over the Pt/C, Ni/C and Ru/C catalysts. They obtained good catalytic performance at 4 h reaction time and reported that at longer time (more than 4 h) the repolymerization reaction becomes predominant over depolymerization. Chen *et al.*^45^ studied the catalytic depolymerization of lignin into bio-oil at a temperature range of 260–320 °C and time of 2–8 h over the Ni/Al-SBA-15 catalyst and found the highest liquefaction (81.4%) at the reaction time of 4 h. They observed that liquefaction and the yield of phenolic monomers decreased as the time further extended from 4 h to 6 h, possibly due to the domination of condensation reactions over the depolymerisation ones. In the current work, as the reaction time 4 h gave the highest light-oil and less solid residue at 290 °C, hence 4 h was selected as the optimal depolymerization time for further investigations.

The effect of reaction temperature on the yield of depolymerized lignin products was investigated in the temperature range of 240–320 °C at 4 h reaction time without catalyst. We note that when reaction temperature was varied from (240 to 320) °C, the pressure value inside the reactor ranged from 64 to 130 bar, which was under the pressure limit (up to 490 bar) of this hydrothermal reactor. In Fig. 5b and Table S2,† it can be clearly seen that at low temperature of 240 and 260 °C, the yields of solid residue were 61.02 wt% and 56.50 wt%, respectively. These results demonstrate that repolymerization reaction could dominate over the depolymerization reaction under low temperature of 260 °C. This observation is also consistent with a previous report,^42^ where alkali lignin was depolymerized in ethanol using CuMgAlOx catalyst at the temperature range of 200–350 °C and it was reported that recondensation reactions dominate at relatively low temperature (<250 °C), while depolymerization reactions become more significant at higher temperature (>250 °C). In the present work, with increasing temperature from 260 to 290 °C, the yields of total bio-oil and light-oil fraction increased to 47.05 and 29.17 wt%, respectively, while the solid residue yield decreased to 41.50 wt%. However, with a further increase in temperature from 290 to 320 °C, the yields of total bio-oil and light-oil were found to decrease while the solid residue yield increased. At higher temperature (>290 °C), the increase in solid residue yield could be attributed to the condensation or repolymerization of reactive species. This phenomenon was also observed in a previous work studied by Feng *et al.*,^19^ who investigated the depolymerization of lignin in ethanol at the temperature range of 250–310 °C and concluded that temperature above 300 °C accelerated the formation of char. In the present study, the FT-IR spectra of solid residues obtained at different temperatures (Fig. S4b†) also gave further evidence that high temperature (>290 °C) favored the condensation or repolymerization of reactive monomers during the KL depolymerization process. From Fig. S4b,† it can be seen that when temperature was increased from 240 °C to 290 °C, the peak intensities of most of the functional groups were found to be decreased substantially. However, at 320 °C, the signal of aromatic ring and hydroxyl groups begin to strong, which could be due to the condensation or repolymerization of reactive monomers. Nakamura *et al.*^46^ investigated the condensation of guaiacol, methylguaiacol and coniferyl alcohols at 250 °C and reported that phenolic aromatic rings and unsaturated side-chain carbons (–CH=CH–) are the most reactive species towards repolymerization. Therefore, considering the maximum yields of bio-oil and light-oil and less solid residue, the temperature 290 °C was chosen as the optimal reaction temperature for the depolymerization of KL in ethanol.

### 3.3 Effect of catalysts

Fig. 5c and Table S2† show the yields of depolymerization products obtained from KL with or without catalyst in ethanol at 290 °C and 4 h. The results indicate that all catalysts exhibited better performance in terms of total bio-oil and light-oil yields compared to the non-catalytic reaction. Without catalyst, the yields of solid residue and light-oil were 41.50 and 29.17 wt%, respectively. When SBA-15 was used as the catalyst, the light-oil yield increased to 32.60 wt%, and the solid residue yield decreased to 38.50 wt%, suggesting that SBA-15 played a role in preventing the repolymerization of reactive intermediates. The better catalytic performance of SBA-15 during lignin depolymerization reaction was also observed in Chen *et al.*^45^ work, where it was reported that SBA-15 effectively suppressed repolymerization reaction due to its large pore size and well-ordered pore structure. When Co/SBA-15 and Mo/SBA-15 are inserted, the product yields are found to be greatly varied. This can be attributed to the deposition of Mo or Co, which provides metal active sites and increases the acidity of SBA-15 (Table 1). However, Co/SBA-15 exhibits a less significant effect in terms of bio-oil yield and reduced solid residue production compared to Mo/SBA-15 catalyst. This is because the acidity of Co/SBA-15 is relatively lower than those of the Mo/SBA-15 catalyst. The presence of metal active sites and the moderate increase in SBA-15 acidity collectively accelerate the cleavage of main linkages (β-O-4′) in lignin, resulting in the production of monomeric products. Interestingly, when Mo/SBA-15 or Co/SBA-15 was replaced by CoMo/SBA-15, the bio-oil and light-oil yields further increased to 56.62 and 48.20 wt%, respectively, while the solid residue yield decreased to 28.50 wt%. This result indicates that the presence of Co and Mo on the SBA-15 framework had a synergistic effect on the hydrogenolysis, hydrogenation and deoxygenation of large lignin fragments, leading to improve bio-oil yield and reduced char formation. Compared to the Mo/SBA-15 and Co/SBA-15 catalyst; CoMo/SBA-15 showed superior performance in terms of reducing solid residue yield. This can be attributed to the larger pore size and higher acid strength in the CoMo/SBA-15 catalyst (Table 1), enabling the adsorption and further degradation of larger lignin fragments into smaller units. This phenomenon is supported by Kim *et al.*^29^ work, where it was reported that the large pore size of SBA-15 has positive effect on the suppression of char. Therefore, CoMo/SBA-15 catalyst was found as optimal catalyst which shows the highest yield of light-oil and lowest yield of solid residue due to presence of metal sites and acid active sites in CoMo/SBA-15 catalyst.

### 3.4 Yield of phenolic monomers in bio-oil

Table 2 shows the yields of phenolic monomers and oligomers present in the light-oil and heavy-oil fractions of bio-oils obtained from KL depolymerization in ethanol at 290 °C for 4 h over different catalysts. It was found that the light-oil fraction of bio-oil contained a large number of monomers (11.56–27.04 wt%), while no significant yields of monomers were identified in the heavy-oil fraction (0.98–1.77 wt%). In the light-oil fraction of non-catalytic bio-oil (at 290 °C and 4 h), the yield of monomers was 11.56 wt%. After the treatment with SBA-15, the yield of monomers increased to 13.67 wt%. The increase of monomers yield with SBA-15 could be related to its well-ordered porous structure which helps to further decomposition of large fragments of lignin into small molecular compounds. The yield of monomers in the light-oil fraction of bio-oils increased again (21.82 wt% and 18.69 wt%) over Mo/SBA-15 and Co/SBA-15 catalysts, respectively (Table 2, entries 3 and 4), indicating the prevention of repolymerization reaction due to metal sites and acid active sites in the Mo/SBA-15 and Co/SBA-15 catalysts. Moreover, in the presence of CoMo/SBA-15 catalyst, the yield of monomeric products also significantly increased to 27.04 wt%, in line with the high yield of light-oil (48.20 wt%) production, which presented a great promotional effect on the depolymerization of KL.

**Table 2:** Yield of monomers and oligomers in bio-oils obtained at 290 °C for 4 h over different catalysts

Entry	Catalyst	Light-oil fraction (wt%)			Heavy-oil fraction (wt%)		
		Monomers (wt%)	Oligomers (wt%)	Total yield (wt%)	Monomers (wt%)	Oligomers (wt%)	Total yield (wt%)
1	Control (290 °C, 4 h)	11.56	17.61	29.17 ± 0.68	0.98	16.90	17.88 ± 0.51
2	SBA-15	13.67	18.93	32.60 ± 0.56	1.20	14.44	15.60 ± 0.33
3	Mo/SBA-15	21.82	21.68	43.50 ± 0.61	1.35	11.61	12.96 ± 0.11
4	Co/SBA-15	18.69	20.21	38.90 ± 0.75	1.46	12.24	13.70 ± 0.43
5	CoMo/SBA-15	27.04	21.16	48.20 ± 0.82	1.77	6.65	8.42 ± 0.17

### 3.5 Bio-oil characterization

#### 3.5.1 GC-MS analysis

**Fig. 6:**
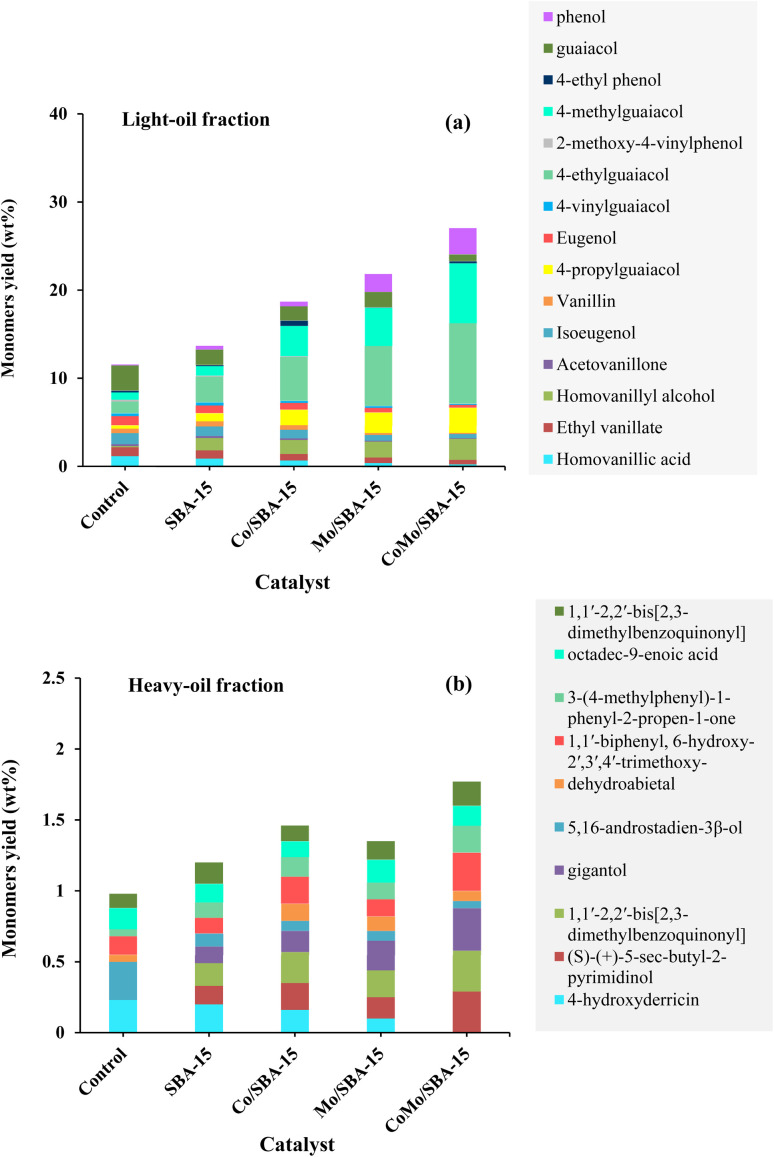
Yield of monomers present in (a) light-oil and (b) heavy-oil fractions of bio-oil obtained without and with catalysts at 290 °C and 4 h.

GC-MS analysis was performed to determine the compositions of monomeric products presented in the light-oil and heavy-oil fractions of bio-oil derived from the non-catalytic and catalytic depolymerization of KL in ethanol at 290 °C for 4 h. Fig. S5–S7† show the mass spectra of some compounds with their standard ones. The GC detected 24 kinds of phenolic monomers. The yields of major monomers and their distribution is shown in Fig. 6a and b. Notably, bio-oils derived from lignin mainly comprised guaiacyl (G)-type phenolics, suggesting that the utilized KL consisted primarily of G-type units. In the light-oil fraction of non-catalytic bio-oil (Fig. 6a), a range of phenolic derivatives, such as guaiacol (2.85 wt%), 4-methylguaiacol (0.83 wt%), 4-ethylguaiacol (1.33 wt%), eugenol (1.04 wt%), isoeugenol (1.25 wt%), homovanillyl alcohol (0.12 wt%) and homovanillic acid (1.16 wt%) were observed. Such compounds are formed from the cleavage of β-O-4 linkages within the guaiacyl units in lignin. Apart from these phenolics, substantial amounts of vanillin and ethyl vanillate were also detected. In a previous study, Kim *et al.*^47^ investigated the effect of sub- and supercritical ethanol on the depolymerization of organosolv lignin. They reported that under supercritical conditions, ethanol acts as a hydrogen donor solvent, facilitating the hydrodeoxygenation and subsequent hydrogenation of oxygenated phenolic monomers. In the present study, when Mo/SBA-15 was used as a catalyst, the yields of eugenol, isoeugenol and homovanillic acid in the light-oil fraction of bio-oil were found to have decreased, while the yields of 4-propylguaiacol, homovanillyl alcohol and 4-ethylguaiacol were increased simultaneously. These findings indicate that 4-propylguaiacol may be the hydrogenated product of eugenol and isoeugenol, whereas homovanillyl alcohol and 4-ethylguaiacol may be the reductive and decarboxylated products of homovanillic acid (HVA). It is expected that the Mo metal sites and acid sites within SBA-15 facilitate the hydrogenation of unsaturated monomers (*e.g.* eugenol and isoeugenol) as well as the reduction or decarboxylation of oxygenated monomers (such as homovanillic acid). In a related study, Luo *et al.*^48^ investigated the influence of Ru metal sites and acid active sites of Al_2_O_3_, TiO_2_ and ZrO_2_ in supercritical ethanol and observed a synergistic effect on the production of phenolic monomers. Chen *et al.*^45^ also reported the positive impact of metal-doped SBA-15 on the production of alkylated phenolic monomers during the treatment of hydrolyzed lignin in ethanol using a Ni/Al-SBA-15 catalyst. It is worth noted that the yield of mono phenol showed an increasing trend under all catalytic conditions. Interestingly, when the bimetallic CoMo/SBA-15 was applied, the yields of 4-ethylguaiacol, 4-methylguaiacol, homovanillyl alcohol and 4-propyl guaiacol were found to increase significantly in the light-oil fraction of bio-oil, which indicates that the CoMo/SBA-15 catalyst enhances the alkylation, hydrogenation and decarboxylation reactions of the unsaturated and oxygenated monomers in the bio-oil, leading to a high yield of selectively alkylated phenolic monomers. This is consistent with the moderate acidity of the CoMo/SBA-15 catalyst, which promotes the production of alkyl phenolic monomers over SBA-15 or Mo/SBA-15 catalysts. The resulting alkyl-guaiacols have potential interest as a green solvent in biorefineries or in higher value-added applications. The phenolic monomers identified in the light-oil fraction of bio-oil were significantly different from the heavy-oil fraction of bio-oils, as shown in Fig. 6b. Moreover, it can be seen that the yield of monomers was very low in the heavy-oil fraction of bio-oil. This finding indicates that heavy-oil fraction of bio-oil was composed of different types of long-chain oligomeric compounds. Among the monomers, gigantol, (S)-(+)-5-sec-butyl-2-pyrimidinol and 1,1′-2,2′-bis[2,3-dimethylbenzoquinonyl were the primary compounds found in the heavy-oil fraction of bio-oil over CoMo/SBA-15 catalyst (Fig. 6b). The overall GC-MS results of this work indicate that the bimetallic CoMo/SBA-15 catalyst is effective for the production of high yield of alkylated phenolics in ethanol under supercritical conditions (290 °C and 4 h).

#### 3.5.2 FT-IR analysis of Kraft lignin and light-oil

**Fig. 7:**
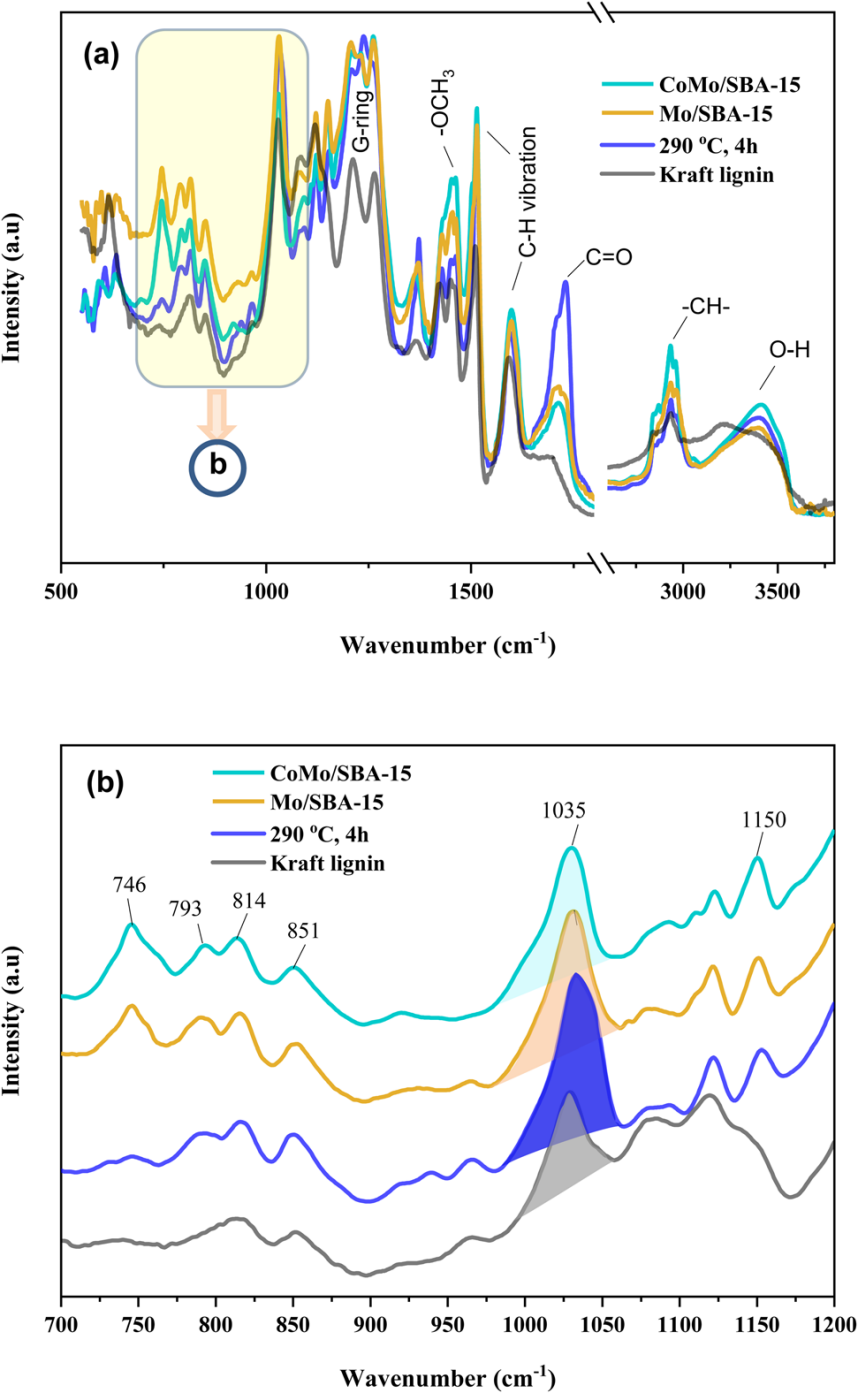
(a) FT-IR spectra of Kraft lignin and light-oils obtained after lignin depolymerization under non-catalytic and catalytic reactions, and (b) expanded (700–1200 cm^−1^) regions of (a).

**Fig. 8:**
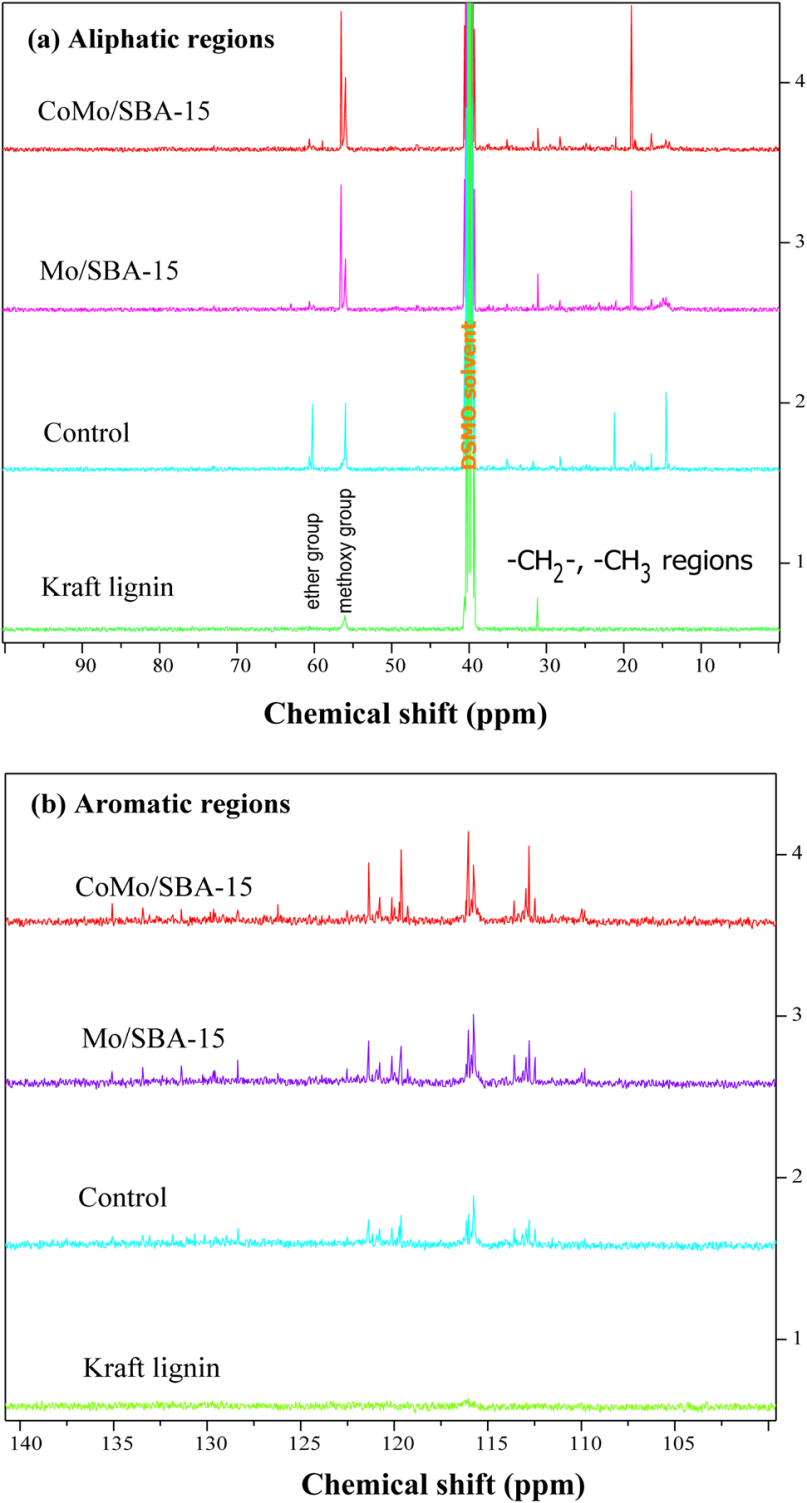
^13^C NMR spectra of Kraft lignin and light-oils obtained at 290 °C for 4 h without (control) and with catalyst. (a) Aliphatic regions and (b) aromatic regions.

The FT-IR analysis was conducted to validate the structural changes occurring before and after the depolymerization of KL. Fig. 7 shows the FT-IR spectra of raw KL and the resulting bio-oils (light-oil fraction) obtained with or without catalysts and the characteristic peaks are summarized in Table S3.† The vibrational band appeared at 3619–3093 cm^−1^ can be attributed to the stretching vibration of the –OH groups in phenolic compounds. The bands at 2928 and 2831 cm^−1^ correspond to the symmetric and asymmetric vibrations of –CH groups attached to aliphatic methyl (–CH_3_), methylene (–CH_2_–) and methoxy groups (–OCH_3_). Clearly, the intensities of these bands were found to be increased in both non-catalytic and catalytic bio-oils compared to raw KL. These results indicate that KL was effectively depolymerized into different types of alkyl substituted phenolic compounds through non-catalytic and catalytic reactions. The peak observed at 1733–1712 cm^−1^ is associated with the carbonyl group (C=O). This peak was also found to have increased in the non-catalytic bio-oil indicating that under supercritical conditions (290 °C and 4 h) without catalyst, ethanol could break the C–O–C and C–C linkages in lignin, resulting in the formation of different phenolic and carbonyl compounds, such as aldehydes, ketones and carboxylic acids. However, with CoMo/SBA-15 catalyst, the carbonyl (C=O) peak intensity was found to be small compared to that of the bio-oil obtained without a catalyst or with Mo/SBA-15. These results are in good agreement with the ^13^C NMR spectra of KL and bio-oils as shown in Fig. 8a, which showed a significant increase in the peak intensities in aliphatic regions (*δ*_C_ = 0–100 ppm) of methyl, methylene and methane groups in the catalytic bio-oil compared to non-catalytic bio-oil or raw KL. Additionally, the non-catalytic bio-oil showed high-intensity peaks of ether or ester groups, which were reduced in the catalytic bio-oils. The presence of metal sites and abundant acid active sites in the CoMo/SBA-15 catalyst may contribute to the enhancement of lignin degradation and the further deoxygenation of carbonyl (C=O) compounds by decarboxylation reactions, leading to the formation of monomers with a lower O content. The bands observed at 1602 and 1512 cm^−1^ can be attributed to the stretching vibration of C–H bonds in the aromatic skeleton, indicating the presence of aromatic compounds. These bands were found to increase in the catalytic bio-oils, which is consistent with the ^13^C NMR results shown in Fig. 8b, where the Mo/SBA-15 and CoMo/SBA-15 catalyzed bio-oils showed high-intensity peaks of aromatic compounds (regions: *δ*_C_ = 100–140 ppm). In Fig. 7, the bands observed at 1270, 1150 and 1118 cm^−1^ were due to the stretching vibration of C–O bonds attached to the guaiacyl ring. Meanwhile the peak appeared at 1035 cm^−1^ is related to the C–O vibration in ether, acid or ester groups. As the non-catalytic bio-oil contained a higher amount of acid and ester compounds, the intensity of the peak at 1035 cm^−1^ was slightly higher compared to that of the bio-oils obtained with Mo/SBA-15 or CoMo/SBA-15 catalyst (Fig. 7b). In addition, the intensities of the peaks at around 860–741 cm^−1^ (assigned to the *para*-substituted aromatic ring) were found to increase in the CoMo/SBA-15 catalyzed bio-oil indicating that CoMo/SBA-15 produced a higher amount of *para*-substituted aromatic monomers in the bio-oil. These results are consistent with the GC-MS result presented in Fig. 6.

#### 3.5.3 2D HSQC NMR analysis of Kraft lignin and light-oil

**Fig. 9:**
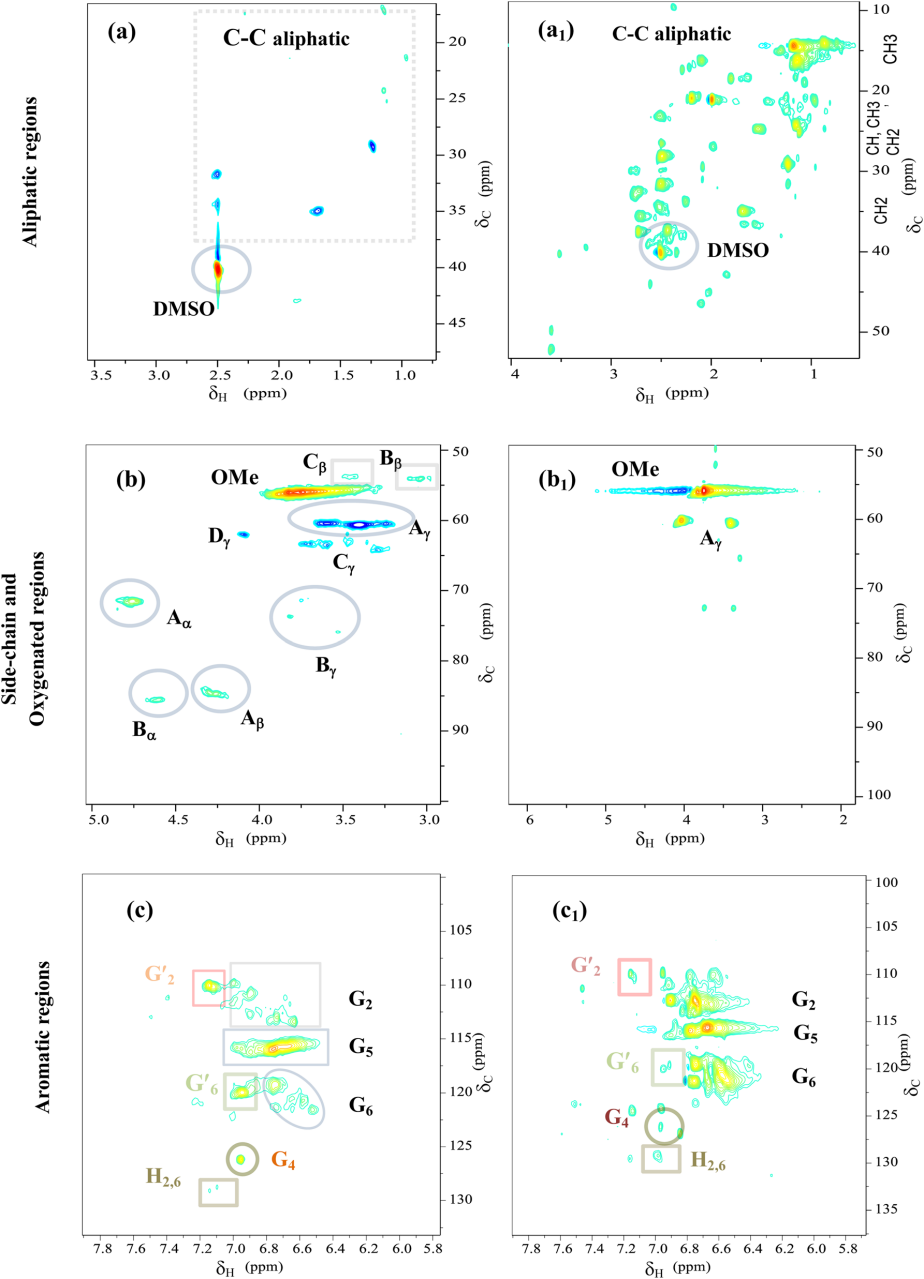
2D HSQC NMR spectra of Kraft lignin (a, b and c) and light-oil (a_1_, b_1_ and c_1_) obtained at 290 °C for 4 h over CoMo/SBA-15 catalyst.

The 2D-HSQC NMR method plays a vital role in elucidating lignin structures and characterizing lignin-derived bio-oil as it provides valuable insights into the structural characteristics of substructures and aromatic units in lignin and bio-oil. To investigate the structural changes in lignin before and after catalytic reaction, 2D HQSC NMR was conducted. Fig. 9 shows the 2D HSQC NMR spectra of raw KL and the bio-oil obtained at 290 °C for 4 h over CoMo/SBA-15 catalyst. The spectra are divided into three regions: the aliphatic hydrocarbon region (*δ*_C_/*δ*_H_ 0–50/0.5–3.5 ppm), the oxygenated side-chain region (*δ*_C_/*δ*_H_ 50–90/3.0–5.0 ppm) and the aromatic region (*δ*_C_/*δ*_H_ 100–135/5.8–7.8 ppm). Peak assignments are provided in Table S4† and Fig. S8–S10 of the ESI,† according to previous studies.^49,50^

In the aliphatic hydrocarbon region (*δ*_C_/*δ*_H_ 0–50/0.5–5.5 ppm), characteristic cross-peaks corresponding to methyl (–CH_3_), methylene (–CH_2_–), and methine (–CH–) groups were found to be appeared to some extend in Kraft lignin (Fig. 9a). However, noticeable differences were observed when comparing these cross-peaks with those of catalytic bio-oil. Most of the cross-peaks observed in the aliphatic region of the catalytic bio-oil were new (Fig. 9(a_1_)), which can be attributed to the formation of phenolic-like ring structures with *ortho*-, *meta*/*para*-methyl, ethyl and propyl substituents. This finding is consistent with the GC-MS results of the bio-oil obtained with the CoMo/SBA-15 catalyst (Fig. 6), which indicates a high content of alkyl-substituted aromatic monomers.

**Fig. 10:**
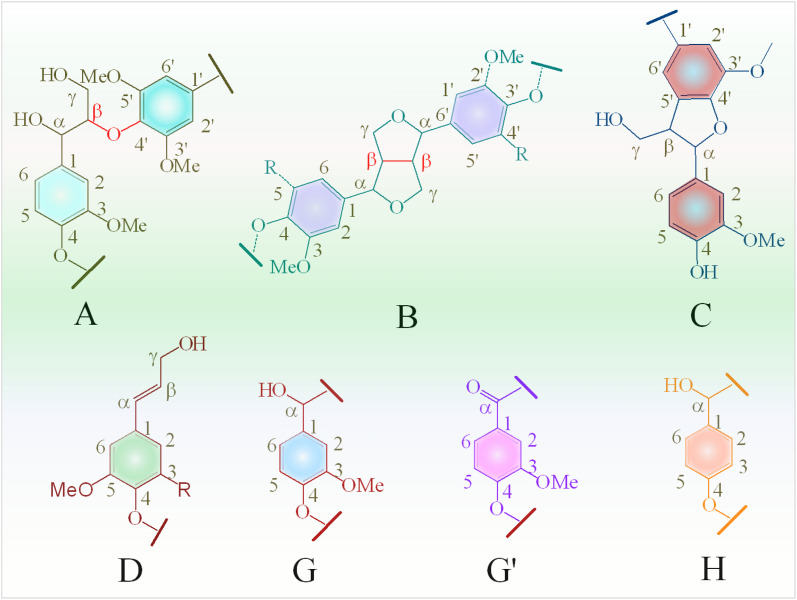
Main substructures in Kraft lignin: (A) β-O-4′ aryl ether linkages; (B) resinol substructures formed by β–β, α-O-γ, and γ-O-α linkages; (C) phenylcoumarane substructures formed by β-5 and α-O-4 linkages; (C) phenylcoumarans; (D) coniferyl alcohol; (G) guaiacyl units; (G′) oxidized guaiacyl units; and (H) *p*-hydroxyphenyl units.

In the oxygenated side-chain region (*δ*_C_/*δ*_H_ 50–100/3.0–5.0 ppm), the presence of inter-unit linkages in KL, namely β-O-4′ aryl ether (A), resinol (β–β′, B), and phenylcoumaran (β-5′, C), was identified through cross-peaks at *δ*_C_/*δ*_H_ 71.58/4.73 (A_α_), 84.68/4.26 (A_β_), 60.55/3.40 and 60.40/3.59 (A_γ_), 85.56/4.60 (B_α_), 54.07/3.04 (B_β_), 75.85/3.53, 73.64/3.83, and 71.44/3.75 (B_γ_), 53.78/3.43 (C_β_) and 63.34/3.72 (C_γ_) (Fig. 9b). The main inter-unit linkages in lignin are illustrated in Fig. 10. After catalytic depolymerization with CoMo/SBA-15 (Fig. 9(b_1_)), the signals corresponding to A_α_, A_β_, B_α,_ B_β,_ B_γ_, C_β_, and C_γ_ linkages in lignin were no longer observed in the bio-oil. This result suggests the near complete cleavage of C–O–C and C–C bonds in KL after depolymerization with the CoMo/SBA-15 catalyst. Moreover, the signal of A_γ_ was still present in the spectra of the catalytic bio-oil, which can be attributed to the production of benzylic alcohol containing an OH group at the γ-position. On the other hand, the signal of methoxy groups (–OMe) (*δ*_C_/*δ*_H_ = 55.83/3.75 ppm) was found to be increased in the catalytic bio-oil. This result corresponds well to the lignin monomer products of GC-MS results (Fig. 6), in which high amount of methoxylated phenols were identified.

In the aromatic region (*δ*_C_/*δ*_H_ 100–135/5.8–7.8 ppm) of the HSQC spectrum of KL (Fig. 9c), guaiacyl (G) units showed prominent and distinct correlation signals at *δ*_C_/*δ*_H_ 109.99/7.14 ppm for oxidized G_2_′, at 110.73/6.90, 112.94/6.74 and 113.38/6.63 ppm for G_2_, at 115.88/6.95 and 115.88/6.75 ppm for G_5_, at 119.41/6.76, 120.74/6.58 and 121.47/6.52 ppm for G_6_, at 120.0/6.95 ppm for G′_6_ and at 126.18/6.95 ppm for G_4_. A minor C_2,6_–H_2,6_ correlation from H units was detected, indicated by a signal at *δ*_C_/*δ*_H_ 129.13/7.14 ppm. However, no signal for S unit was observed in the HSQC spectrum of lignin, suggesting that the KL used belonged to GH-type lignin. Upon the treatment of KL with a 10 wt% loading of CoMo/SBA-15 at 290 °C for 4 h in ethanol, a brown oily lignin product was obtained, exhibiting an increase in the intensity of G-type units (Fig. 9c_1_). These results are consistent with the FT-IR results (Fig. 7a), where the intensity of the G ring band in KL exhibited a lower peak intensity compared to that of the catalytic bio-oils.

#### 3.5.4 Elemental analysis of heavy-oils

Elemental analysis was conducted on raw KL and the bio-oils obtained with or without catalysts and the results are summarized in Table S5.† It can be seen that KL has higher oxygen (O), nitrogen (N), and sulfur (S) contents than KL-derived bio-oils. For example, the O, N and S contents of KL were 30.09%, 0.65% and 1.48%, respectively. After KL depolymerization, these values decreased to 26.23%, 0.39% and 1.05% in the heavy-oil fraction of non-catalytic bio-oil, resulting in a relatively high HHV value of 28.44 MJ kg^−1^ (Table S5,† entry 2). When Mo/SBA-15 and CoMo/SBA-15 catalysts were used (Table S5,† entries 3 and 4), the O, N and S content further decreased, while the C and H content increased. Consequently, the HHV values also increased to 29.24 MJ kg^−1^ and 29.75 MJ kg^−1^, respectively. For comparison, the composition of C, H, N, S and O in the light-oil fraction obtained with the CoMo/SBA-15 catalyst was also determined (Table S5,† entry 5). The light-oil fraction showed a higher C (71.23%) and H (8.31%) content, and lower O (20.16%) and S (0.07%) contents, resulting in a higher HHV value of 32.60 MJ kg^−1^ compared to that of the heavy-oil fraction (29.75 MJ kg^−1^). These results reveal that CoMo/SBA-15 is an excellent catalyst for hydrogenation, hydrodesulfurization and deoxygenation during the lignin depolymerization in supercritical ethanol.

**Fig. 11:**
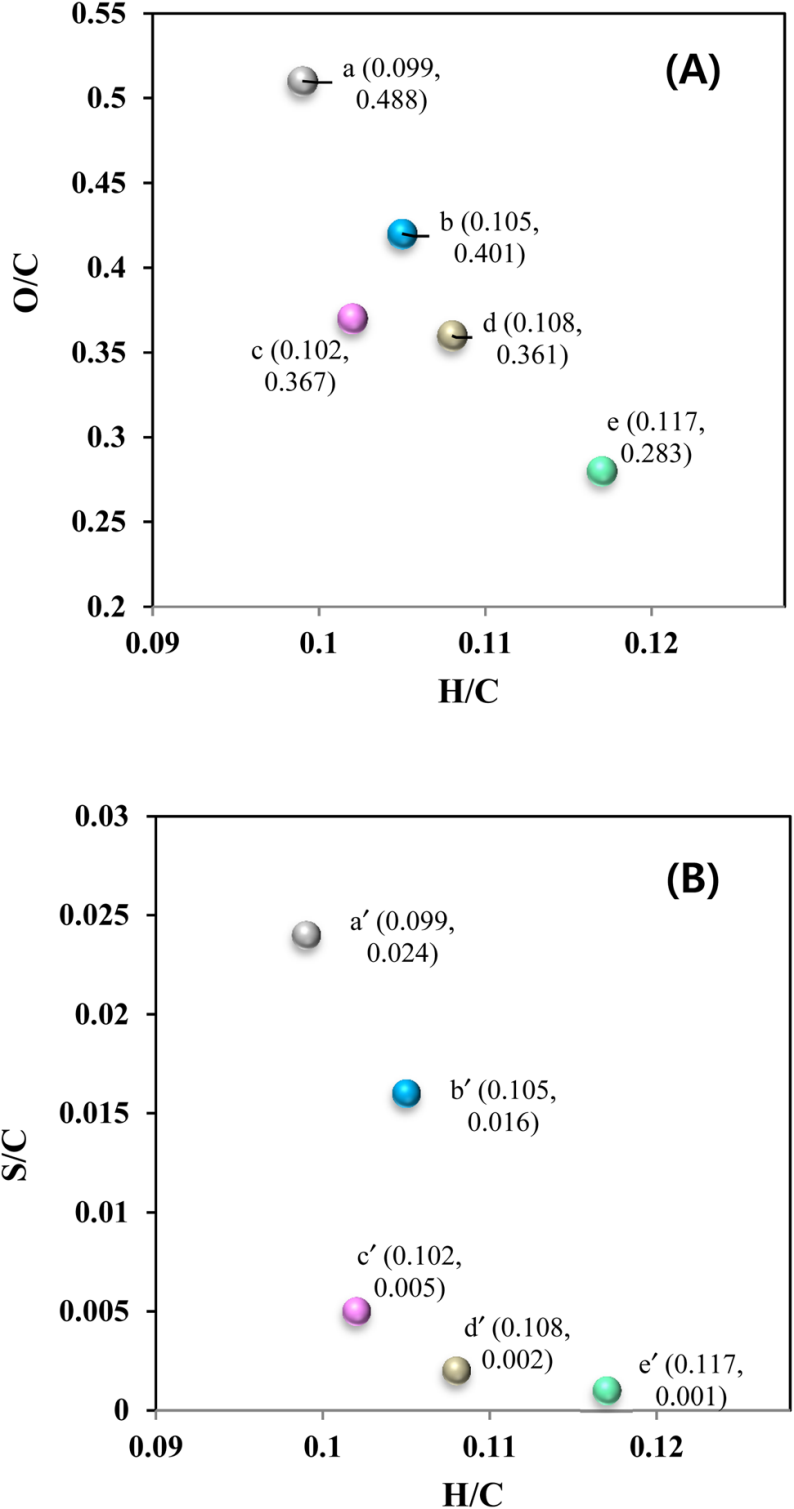
Van Krevelen diagram of Kraft lignin and heavy-oil fractions (A and B). The O/C *vs.* H/C, and S/C *vs.* H/C ratios of (a and a′) Kraft lignin and heavy-oil fractions obtained at (b and b′) 290 °C and 4 h, (c and c′) 290 °C, 4 h and Mo/SBA-15 catalyst, (d and d′) 290 °C, 4 h and CoMo/SBA-15 catalyst, and (e and e′) light-oil fraction obtained at 290 °C, 4 h and CoMo/SBA-15 catalyst.

The O/C and H/C ratios of KL and the heavy-oil fraction of bio-oils are shown in Fig. 11A, while the S/C and H/C ratios are shown in Fig. 11B. It can be seen that the O/C and H/C ratios of KL were 0.488 and 0.099, respectively. After the depolymerization of KL at 290 °C for 4 h (control experiment), the O/C ratio decreased to 0.401, and the H/C ratio increased to 0.105. Notably, in the presence of the CoMo/SBA-15 catalyst, the O/C ratio decreased and the H/C ratio increased significantly. This finding indicates that the bimetallic CoMo/SBA-15 catalyst could enhance the hydrogenation of unsaturated bonds in lignin and lignin-derived depolymerized products, leading to an increased H/C ratio in the heavy-oil fraction. Furthermore, the O/C and H/C ratios of light-oil fraction obtained with the CoMo/SBA-15 catalyst were also measured. It is noteworthy that the CoMo/SBA-15 catalyst yielded a light-oil with a higher H/C ratio and a lower O/C ratio, as well as a higher HHV value. On the other hand, the S/C and H/C ratios of KL were 0.024 and 0.099, respectively (Fig. 11B). However, the S/C ratio was found to be decreased in the heavy-oil and the light-oil fractions of bio-oil to 0.12 and 0.001, respectively, in the presence of CoMo/SBA-15 catalyst. The above findings suggest that the CoMo/SBA-15 catalyst promotes the hydrogenolysis of lignin into various monomeric products and the further hydrogenation and deoxygenation of those products, resulting in the formation of saturated phenolic monomers, facilitated by the highly dispersed metal sites and acid active sites of the CoMo/SBA-15 catalyst.

### 3.6 Reaction route and mechanism

**Scheme 1:**
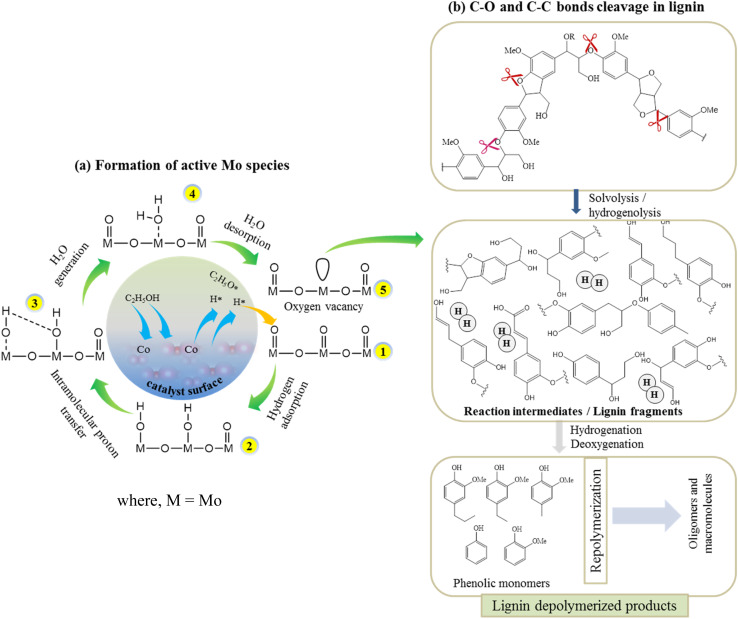
Possible reaction routes during lignin depolymerization over the CoMo/SBA-15 catalyst.

According to the XPS results, the Co existed in the form of (+3 and +2) oxidation states while Mo existed as +6 and +5 oxidation states (Fig. 3). The variable electronic valences of Co and Mo-based catalysts make them promising catalyst in various heterogeneous catalytic reactions. In this study, to gain a better insight into the interaction between KL and bifunctional CoMo/SBA-15 catalyst, the possible catalytic reaction mechanism is proposed in Scheme 1. Under supercritical condition, lignin was firstly decomposed into different reaction intermediates or macromolecular fragments by the solvolysis (*e.g.* ethanolysis). Subsequently, the macromolecular fragments may undergo various reactions such as hydrogenolysis, dehydration and decarboxylation, leading to the production of different derivatives of phenolic compounds. In the presence of catalyst (*i.e.* CoMo/SBA-15), the alcoholic solvent expected to produce active H* and ethoxy species (Scheme 1a). At the same time, the ethoxy group may activate saturated Mo species, resulting in active Mo sites. On the other hand, the dissociated active H species adsorbed on the adjacent terminal oxygen of MoOx species to form hydroxyl groups. Thereafter, an intramolecular proton transfer reaction may undergo to generate H_2_O molecule. Finally, the generated H_2_O species removed from the Mo surface to form active Mo sites with oxygen vacancy (*i.e.* unsaturated coordination). The active Mo species with oxygen vacancy then attacked the C–O bonds of macromolecular fragments (Scheme 1b) to generate intermediates products and further the intermediate products attacked by the many dissociative hydrogen species and transformed into phenolic derivatives through unsaturated side-chain hydrogenation and deoxygenation/dehydration reactions. The formation of unsaturated coordination in Mo species was also confirmed in Guo *et al.*^51^ work, who investigated catalytic depolymerization of KL and hydrodeoxygenation of anisole (lignin-derived model compound) over MoOx/ZIF-8@ZIF-67 catalyst. They reported the formation of unsaturated coordination site (oxygen vacancy) in Mo catalyst and further the adsorption of C–O bonds of KL-derived intermediates or anisole onto the active Mo species. The overall results reveal that under supercritical ethanol, the main mechanisms for the formation of different aromatic phenolic monomers over CoMo/SBA-15 catalyst were solvolysis/hydrogenolysis, side-chain hydrogenation and deoxygenation (through dehydration) reactions.

## 4. Conclusion

In this contribution, we prepared monometallic Mo/SBA-15, Co/SBA-15 and bimetallic CoMo/SBA-15 catalysts *via* the wet impregnation method for the depolymerization of KL in supercritical ethanol. Our results showed that the CoMo/SBA-15 catalyst outperformed the Mo/SBA-15 and Co/SBA-15 catalysts in terms of depolymerization efficiency and the selective production of phenolic monomers from lignin. The presence of metal active sites in the CoMo catalyst and acid active sites in the SBA-15 support synergistically contribute to the cleavage of C–O–C and C–C bonds in lignin, resulting in a maximum phenolic monomer yield of 27.04 wt% at 290 °C and 4 h. We found that KL can be effectively depolymerized in supercritical ethanol with the CoMo/SBA-15 catalyst *via* hydrogenolysis, hydrogenation and subsequent deoxygenation reactions. GC-MS and FT-IR analyses of bio-oil showed that the absence of a catalyst led to the formation of carbonyl compounds such as vanillin, ethyl vanillate and homovanillic acid in ethanol. In contrast, more alkylated phenols were produced in ethanol over CoMo/SBA-15 catalyst. The major phenolic monomers identified in the catalytic bio-oil were 4-methylguaiacol, 4-ethylguaiacol and 4-propyl guaiacol. Furthermore, elemental analysis confirmed a reduction in the O content of the catalytic bio-oil, indicating the excellent hydrogenation and reductive catalytic activity of the CoMo/SBA-15 catalyst in ethanol.

## Conflicts of interest

There are no conflicts to declare.

## Supplementary Material

RA-013-D3RA05018A-s001

## References

[cit1] Rana M., Nshizirungu T., Park J.-H. (2021). Synergistic Effect of Water-Ethanol-Formic Acid for the Depolymerization of Industrial Waste (Black Liquor) Lignin to Phenolic Monomers. Biomass Bioenergy.

[cit2] Rana M., Nshizirungu T., Park J. H. (2022). Effect of Simultaneous Use of Microwave and Ultrasound Irradiation on the Sulfuric Acid Hydrolysis Lignin (SAHL) Depolymerization. Sustainable Energy Fuels.

[cit3] Rana M., Taki G., Islam M. N., Agarwal A., Jo Y. T., Park J. H. (2019). Effects of Temperature and Salt Catalysts on Depolymerization of Kraft Lignin to Aromatic Phenolic Compounds. Energy Fuels.

[cit4] Agarwal A., Rana M., Park J. H. (2018). Advancement in Technologies for the Depolymerization of Lignin. Fuel Process. Technol..

[cit5] Nde D. B., Muley P. D., Sabliov C. M., Nokes S. E., Boldor D. (2021). Microwave Assisted Pyrolysis of Kraft Lignin in Single Mode High-Q Resonant Cavities : Degradation Kinetics , Product Chemical Composition , and Numerical Modeling. Energy Convers. Manage..

[cit6] Peng M., Muraishi T., Hou X., Zhao M., Kamiya K., Qian E. W. (2023). Oxidative Depolymerization of Lignin to Vanillin and Lactic Acid in an Aqueous Solution. Fuel.

[cit7] Shin H. Y., Jo S. M., Kim S. S. (2022). Oxidative Depolymerization of Kraft Lignin Assisted by Potassium Tert-Butoxide and Its Effect on Color and UV Absorption. Ind. Crops Prod..

[cit8] Zhang J., Zhu X., Xu X., Sun Q., Wei L., Li K., Zhai S., An Q. (2022). Journal of Environmental Chemical Engineering Cooperative Catalytic Effects between Aqueous Acidic Ionic Liquid Solutions and Polyoxometalate-Ionic Liquid in the Oxidative Depolymerization of Alkali Lignin. J. Environ. Chem. Eng..

[cit9] Hao G., Liu H., Chang Z., Song K., Yang X., Ma H., Wang W. (2022). Catalytic Depolymerization of the Dealkaline Lignin over Co–Mo–S Catalysts in Supercritical Ethanol. Biomass Bioenergy.

[cit10] Zhang X., Li W., Wang J., Zhang B., Guo G., Shen C., Jiang Y. (2022). Depolymerization of Kraft Lignin into Liquid Fuels over a WO3 Modified Acid-Base Coupled Hydrogenation Catalyst. Fuel.

[cit11] Kumar A., Yadav P., Reddy S. N. (2023). Catalytic (Copper) Hydrothermal Liquefaction for Lignin to Produce High Quality Bio-Oil and Nano Cu Carbon Hybrids Material. Chem. Eng. Sci..

[cit12] Feng L., Li X., Wang Z., Liu B. (2021). Catalytic Hydrothermal Liquefaction of Lignin for Production of Aromatic Hydrocarbon over Metal Supported Mesoporous Catalyst. Bioresour. Technol..

[cit13] Klein I., Saha B., Abu-Omar M. M. (2015). Lignin Depolymerization over Ni/C Catalyst in Methanol, a Continuation: Effect of Substrate and Catalyst Loading. Catal. Sci. Technol..

[cit14] Warner G., Hansen T. S., Riisager A., Beach E. S., Barta K., Anastas P. T. (2014). Depolymerization of Organosolv Lignin Using Doped Porous Metal Oxides in Supercritical Methanol. Bioresour. Technol..

[cit15] Yan F., Ma R., Ma X., Cui K., Wu K., Chen M., Li Y. (2017). Ethanolysis of Kraft Lignin to Platform Chemicals on a MoC1-x/Cu-MgAlOz Catalyst. Appl. Catal., B.

[cit16] Baxter N. C., Wang Y., Huang H., Liao Y., Barnett H., Zhao Y., Wang S. (2021). Kraft Lignin Ethanolysis over Zeolites with Different Acidity and Pore Structures for Aromatics Production. Catalysts.

[cit17] Liu Q., Li P., Liu N., Shen D. (2017). Lignin Depolymerization to Aromatic Monomers and Oligomers in Isopropanol Assisted by Microwave Heating. Polym. Degrad. Stab..

[cit18] Kong L., Liu C., Gao J., Wang Y., Dai L. (2019). Efficient and Controllable Alcoholysis of Kraft Lignin Catalyzed by Porous Zeolite-Supported Nickel-Copper Catalyst. Bioresour. Technol..

[cit19] Feng L., Li X., Wang Z., Liu B. (2021). Catalytic Hydrothermal Liquefaction of Lignin for Production of Aromatic Hydrocarbon over Metal Supported Mesoporous Catalyst. Bioresour. Technol..

[cit20] Bui V. N., Laurenti D., Afanasiev P., Geantet C. (2011). Hydrodeoxygenation of Guaiacol with CoMo Catalysts. Part I: Promoting Effect of Cobalt on HDO Selectivity and Activity. Appl. Catal., B.

[cit21] Dou X., Jiang X., Li W., Zhu C., Liu Q., Lu Q., Zheng X., Chang H. M., Jameel H. (2020). Highly Efficient Conversion of Kraft Lignin into Liquid Fuels with a Co-Zn-Beta Zeolite Catalyst. Appl. Catal., B.

[cit22] Wang Y., Tang Z., Chen M., Zhang J., Shi J., Wang C., Yang Z., Wang J. (2020). Effect of Mo Content in Mo/Sepiolite Catalyst on Catalytic Depolymerization of Kraft Lignin under Supercritical Ethanol. Energy Convers. Manage..

[cit23] Zhang L., Shang N., Gao S., Wang J., Meng T., Du C., Shen T., Huang J., Wu Q., Wang H., Qiao Y., Wang C., Gao Y., Wang Z. (2020). Atomically Dispersed Co Catalyst for Efficient Hydrodeoxygenation of Lignin-Derived Species and Hydrogenation of Nitroaromatics. ACS Catal..

[cit24] Yang Y., Lv G., Deng L., Lu B., Li J., Zhang J., Shi J., Du S. (2017). Renewable Aromatic Production through Hydrodeoxygenation of Model Bio-Oil over Mesoporous Ni/SBA-15 and Co/SBA-15. Microporous Mesoporous Mater..

[cit25] Pu J., Nguyen T. S., Leclerc E., Lorentz C., Laurenti D., Pitault I., Tayakout-Fayolle M., Geantet C. (2019). Lignin Catalytic Hydroconversion in a Semi-Continuous Reactor: An Experimental Study. Appl. Catal., B.

[cit26] Pu J., Laurenti D., Geantet C., Tayakout-Fayolle M., Pitault I. (2020). Kinetic Modeling of Lignin Catalytic Hydroconversion in a Semi-Batch Reactor. Chem. Eng. J..

[cit27] Hao G., Liu H., Chang Z., Song K., Yang X., Ma H., Wang W. (2022). Catalytic Depolymerization of the Dealkaline Lignin over Co–Mo–S Catalysts in Supercritical Ethanol. Biomass Bioenergy.

[cit28] Jongerius A. L., Bruijnincx P. C. A., Weckhuysen B. M. (2013). Liquid-Phase Reforming and Hydrodeoxygenation as a Two-Step Route to Aromatics from Lignin. Green Chem..

[cit29] Kim J. Y., Park S. Y., Choi I. G., Choi J. W. (2018). Evaluation of RuxNi1-x/SBA-15 Catalysts for Depolymerization Features of Lignin Macromolecule into Monomeric Phenols. Chem. Eng. J..

[cit30] Lu X., Guo H., Chen J., Wang D., Lee A. F., Gu X. (2022). Selective Catalytic Transfer Hydrogenation of Lignin to Alkyl Guaiacols Over NiMo/Al-MCM-41. ChemSusChem.

[cit31] Klamrassamee T., Laosiripojana N., Cronin D., Moghaddam L., Zhang Z., Doherty W. O. S. (2015). Effects of Mesostructured Silica Catalysts on the Depolymerization of Organosolv Lignin Fractionated from Woody Eucalyptus. Bioresour. Technol..

[cit32] Ma R., Hao W., Ma X., Tian Y., Li Y. (2014). Catalytic Ethanolysis of Kraft Lignin into High-Value Small-Molecular Chemicals over a Nanostructured α-Molybdenum Carbide Catalyst. Angew. Chem., Int. Ed..

[cit33] Biswas B., Kumar A., Krishna B. B., Baltrusaitis J., Adhikari S., Bhaskar T. (2022). Catalytic Depolymerization of Lignin for the Selective Production of Phenolic Monomers over Cobalt-Supported Calcium Catalysts. Energy Fuels.

[cit34] Chen M., Hao W., Ma R., Ma X., Yang L., Yan F., Cui K., Chen H., Li Y. (2017). Catalytic Ethanolysis of Kraft Lignin to Small-Molecular Liquid Products over an Alumina Supported Molybdenum Nitride Catalyst. Catal. Today.

[cit35] Dou X., Li W., Zhu C. (2021). Catalytic Hydrotreatment of Kraft Lignin into Liquid Fuels over Porous ZnCoOx Nanoplates. Fuel.

[cit36] Ghoreishi S., Barth T., Derribsa H. (2019). Formic Acid Assisted Liquefaction of Lignin in Water and Ethanol, Investigated for a 0.025 and a 5 L Batch Reactor: Comparison of Yields and Compositions of the Products. Biomass Bioenergy.

[cit37] Rana M., Islam M. N., Agarwal A., Taki G., Park S. J., Dong S., Jo Y. T., Park J. H. (2018). Production of Phenol-Rich Monomers from Kraft Lignin Hydrothermolysates in Basic-Subcritical Water over MoO3/SBA-15 Catalyst. Energy Fuels.

[cit38] Zhao D., Feng J., Huo Q., Melosh N., Fredrickson G. H., Chmelka B. F., Stucky G. D. (1998). Triblock Copolymer Syntheses of Mesoporous Silica with Periodic 50 to 300 Angstrom Pores. Science.

[cit39] Huang J., Qian W., Ma H., Zhang H., Ying W. (2017). Highly Selective Production of Heavy Hydrocarbons over Cobalt-Graphene-Silica Nanocomposite Catalysts. RSC Adv..

[cit40] Smyrnioti M., Ioannides T. (2017). Synthesis of Cobalt-Based Nanomaterials from Organic Precursors. Cobalt.

[cit41] Jiménez-Morales I., Vila F., Mariscal R., Jiménez-López A. (2012). Hydrogenolysis of Glycerol to Obtain 1,2-Propanediol on Ce-Promoted Ni/SBA-15 Catalysts. Appl. Catal., B.

[cit42] Huang X., Korányi T. I., Boot M. D., Hensen E. J. M. (2014). Catalytic Depolymerization of Lignin in Supercritical Ethanol. ChemSusChem.

[cit43] Roberts V. M., Stein V., Reiner T., Lemonidou A., Li X., Lercher J. A. (2011). Towards Quantitative Catalytic Lignin Depolymerization. Chem. - Eur. J..

[cit44] Bartolomei E., Le Brech Y., Gadiou R., Bertaud F., Leclerc S., Vidal L., Le Meins J. M., Dufour A. (2021). Depolymerization of Technical Lignins in Supercritical Ethanol: Effects of Lignin Structure and Catalyst. Energy Fuels.

[cit45] Chen P., Zhang Q., Shu R., Xu Y., Ma L., Wang T. (2017). Catalytic Depolymerization of the Hydrolyzed Lignin over Mesoporous Catalysts. Bioresour. Technol..

[cit46] Nakamura T., Kawamoto H., Saka S. (2007). Condensation Reactions of Some Lignin Related Compounds at Relatively Low Pyrolysis Temperature. J. Wood Chem. Technol..

[cit47] Kim J. Y., Oh S., Hwang H., Cho T. S., Choi I. G., Choi J. W. (2013). Effects of Various Reaction Parameters on Solvolytical Depolymerization of Lignin in Sub- and Supercritical Ethanol. Chemosphere.

[cit48] Luo B., Huang Z., Shu R., Cheng Y., Tian Z., Wang C., Chen Y. (2021). Effects of Metal Sites and Acid Sites on the Hydrogenolysis of Cornstalks in Supercritical Ethanol during Lignin-First Fractionation. Sustainable Energy Fuels.

[cit49] Mattsson C., Andersson S. I., Belkheiri T., Åmand L. E., Olausson L., Vamling L., Theliander H. (2016). Using 2D NMR to Characterize the Structure of the Low and High Molecular Weight Fractions of Bio-Oil Obtained from LignoBoost™ Kraft Lignin Depolymerized in Subcritical Water. Biomass Bioenergy.

[cit50] Wen J. L., Yuan T. Q., Sun S. L., Xu F., Sun R. C. (2014). Understanding the Chemical Transformations of Lignin during Ionic Liquid Pretreatment. Green Chem..

[cit51] Guo G., Chen D., Ahmed T., Dou X., Chen K., Li W. (2021). Catalytic Depolymerization of Kraft Lignin towards Liquid Fuels over Bifunctional Molybdenum Oxide Based Supported Catalyst. Fuel.

