# Supplementary material for: Catalytic depolymerization of Kraft lignin to high yield alkylated-phenols over CoMo/SBA-15 catalyst in supercritical ethanol
Source: RSC Adv. 2023 Oct 13;13(43):30022–39. doi: 10.1039/d3ra05018a (PMC10570907; doi:10.1039/d3ra05018a)
Supplement: RA-013-D3RA05018A-s001 [file RA-013-D3RA05018A-s001.pdf]

Electronic Supplementary Material (ESI) for RSC Advances.
This journal is © The Royal Society of Chemistry 2023

Supplementary file for

Catalytic depolymerization of Kraft lignin to high yield alkylated–phenols over CoMo/SBA-15 catalyst in supercritical ethanol

Masud Rana, Shubho Ghosh, Theoneste Nshizirungu and Jeong-Hun Park*

Department of Environment and Energy Engineering, Chonnam National University, Gwangju, 61186, South Korea.

Corresponding author: Tel: 82-62-530-1855, Fax: 82-62-530-1859;

E-mail: parkjeo1@jnu.ac.kr

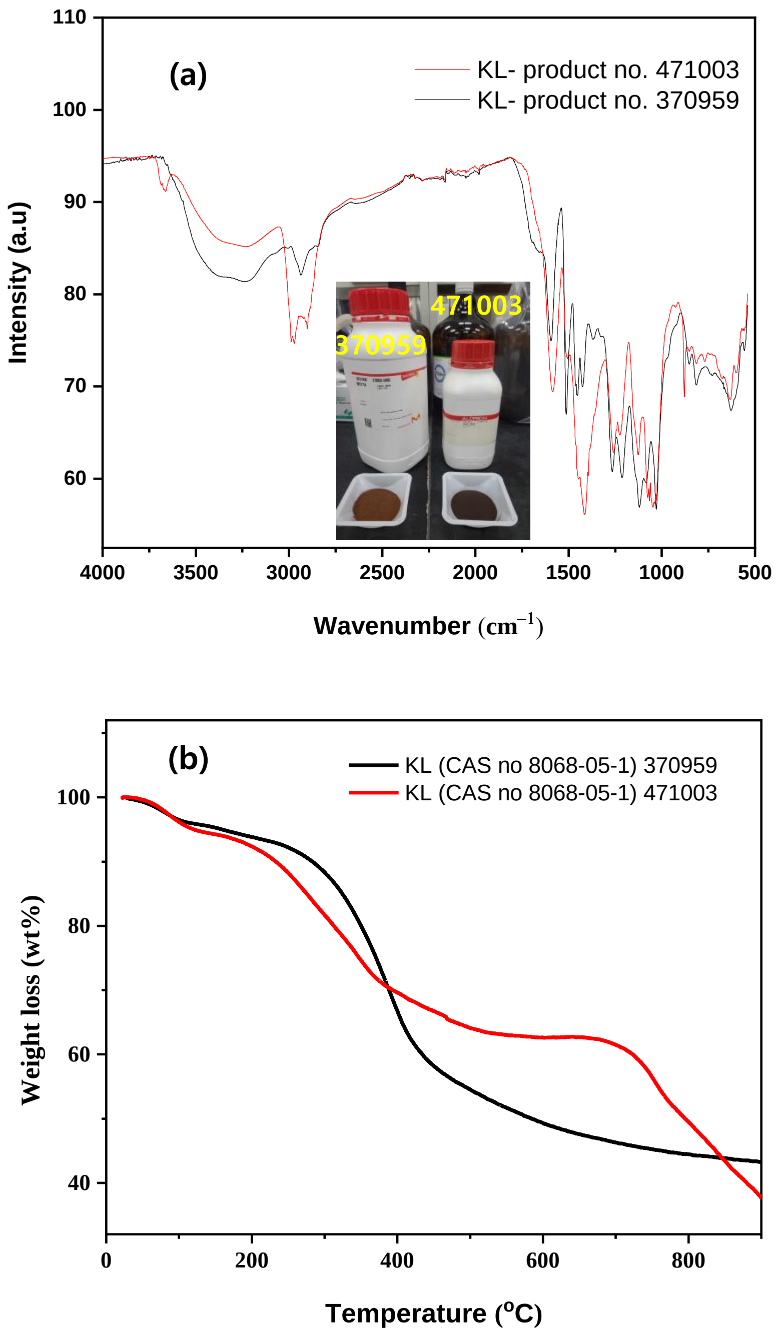

Fig. S1 (a) FT-IR spectra and (b) thermogravimetric (TG) analyses of commercially available KL (Sigma-Aldrich, USA) with the product number 370959 and 471003.

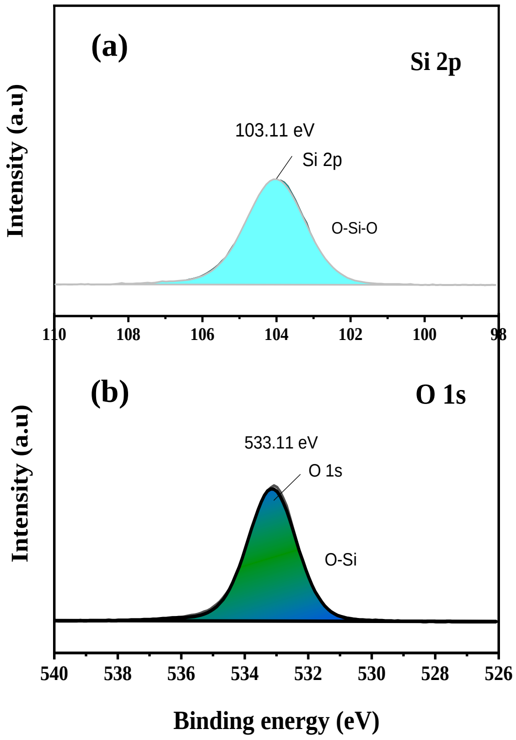

Fig. S2 Core level spectra of (a, b) Si 2p and O 1s in SBA-15.

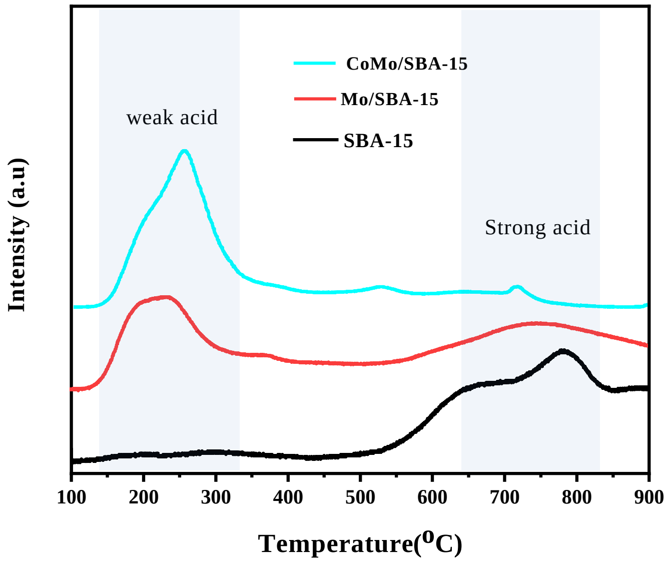

Fig. S3 NH_3–TPD spectra of SBA-15, Mo/SBA-15 and CoMo/SBA-15 catalysts.

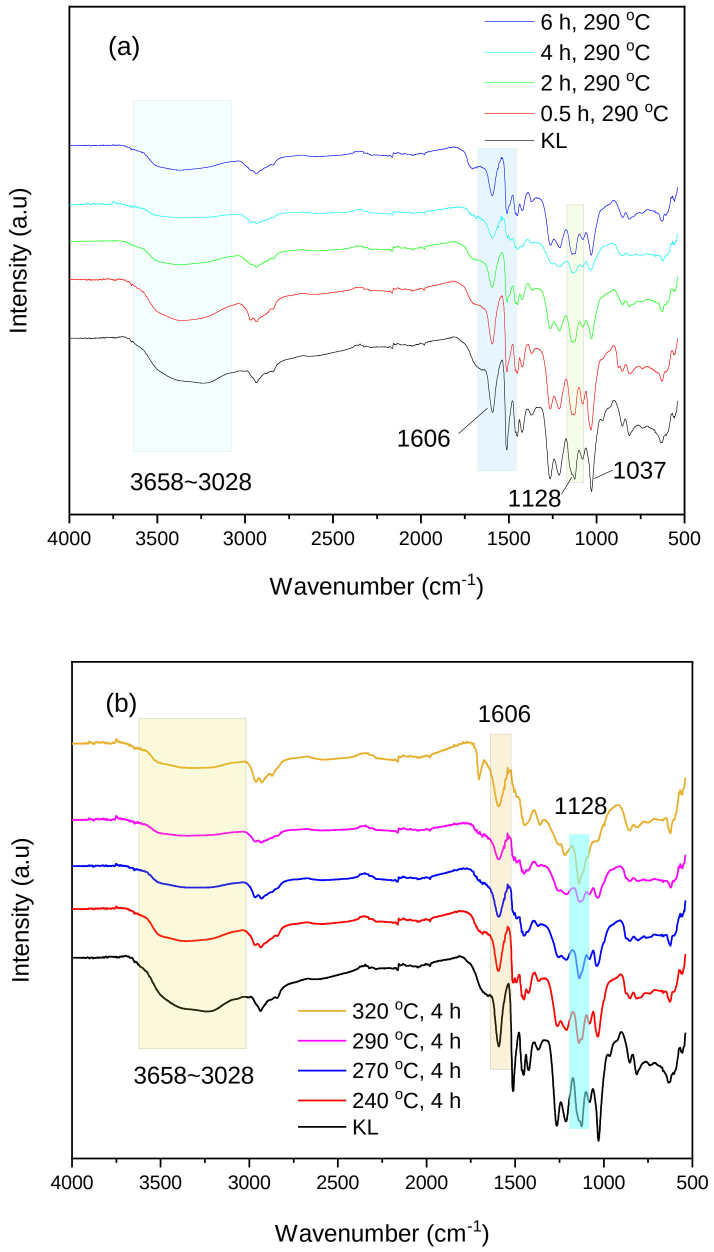

Fig. S4 FTIR spectra of solid residues obtained at different times (a) and temperatures (b).

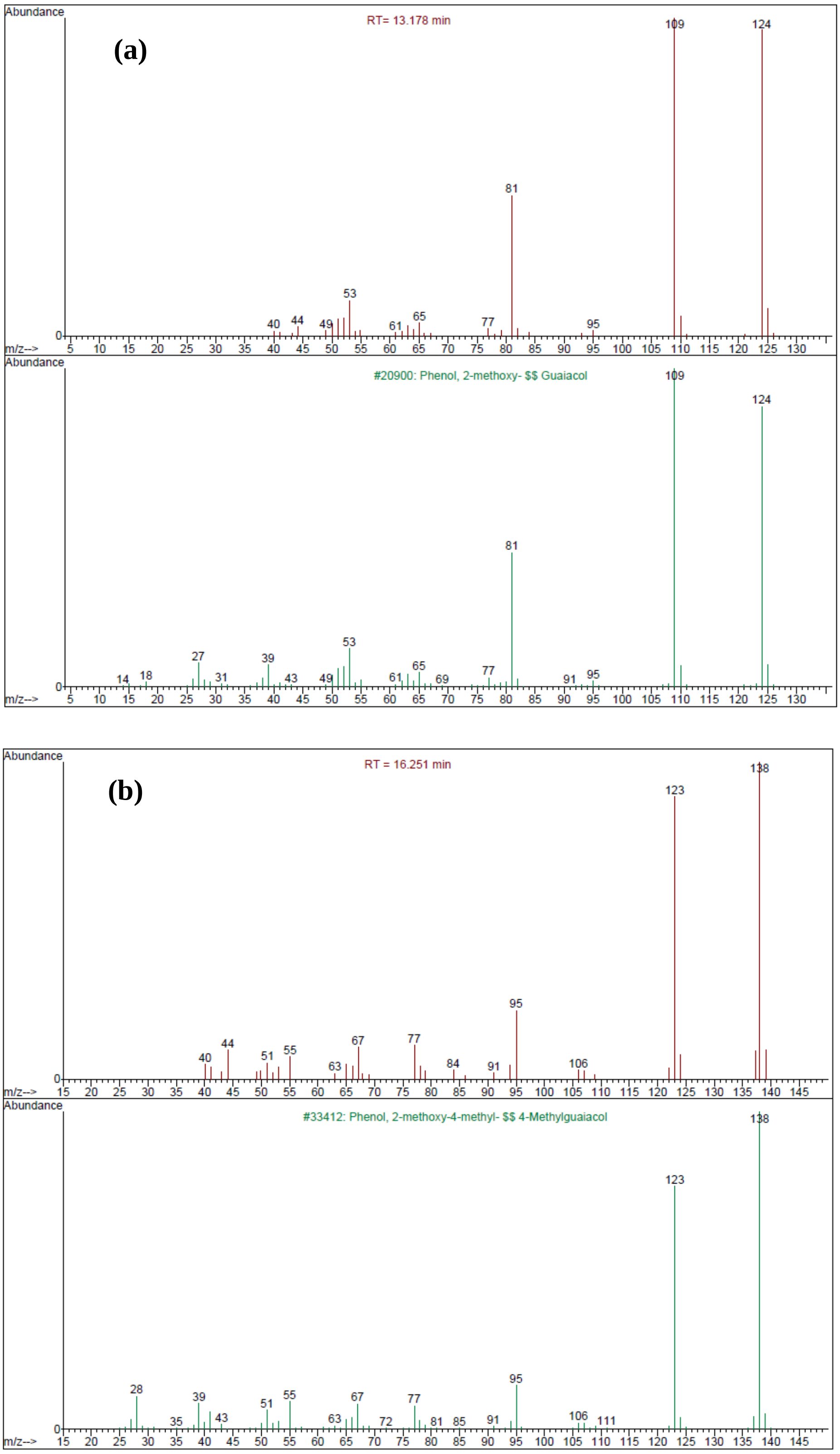

Fig. S5 (a) Mass spectra from the peak13.20 and the standard guaiacol. (b) Mass spectra from the peak 16.25 and the standard 4-methylguaiacol.

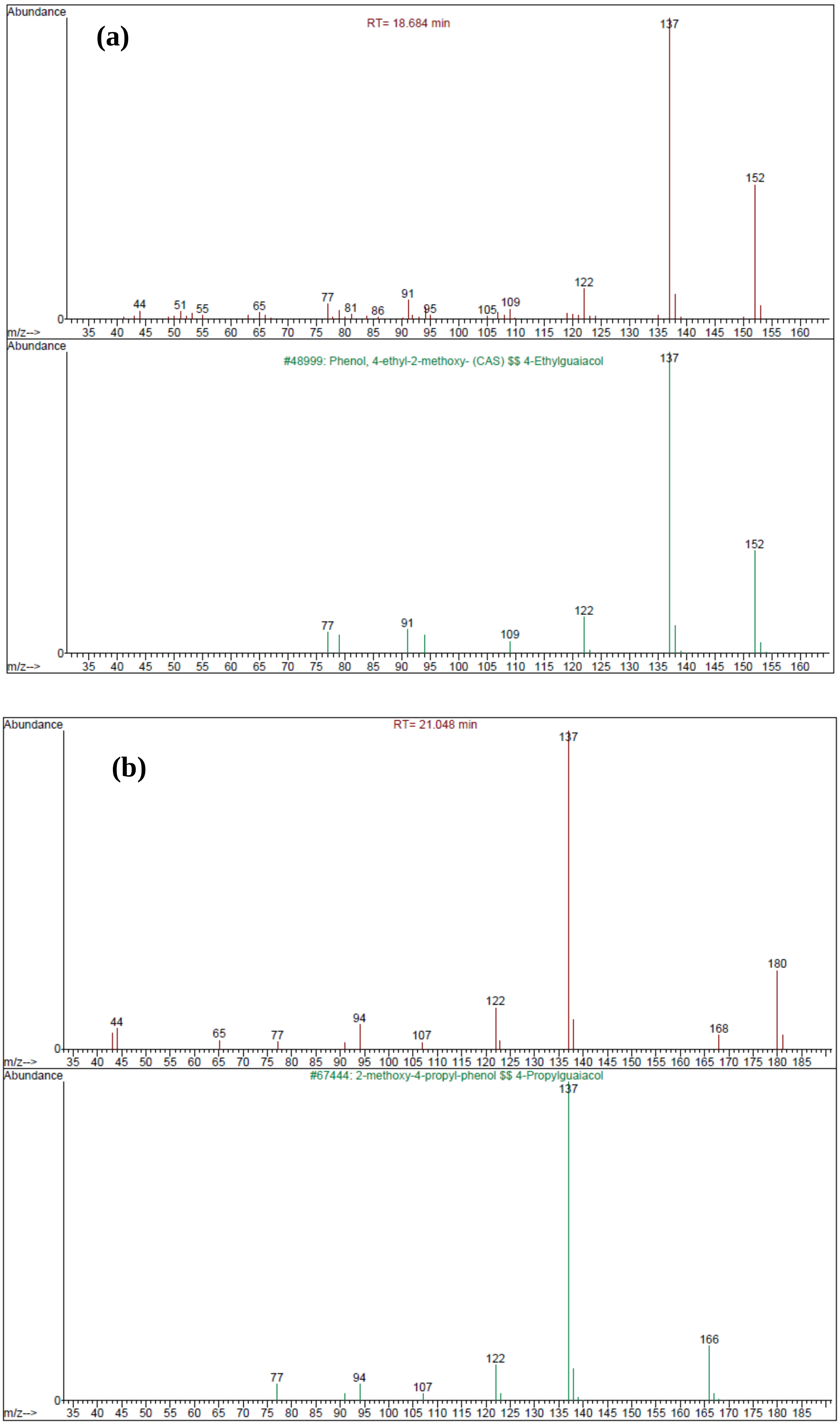

Fig. S6 (a) Mass spectra from the peak 18.67 and the standard 4-ethylguaiacol. (b) Mass spectra from the peak 21.05 and the standard 4-propylguaiacol.

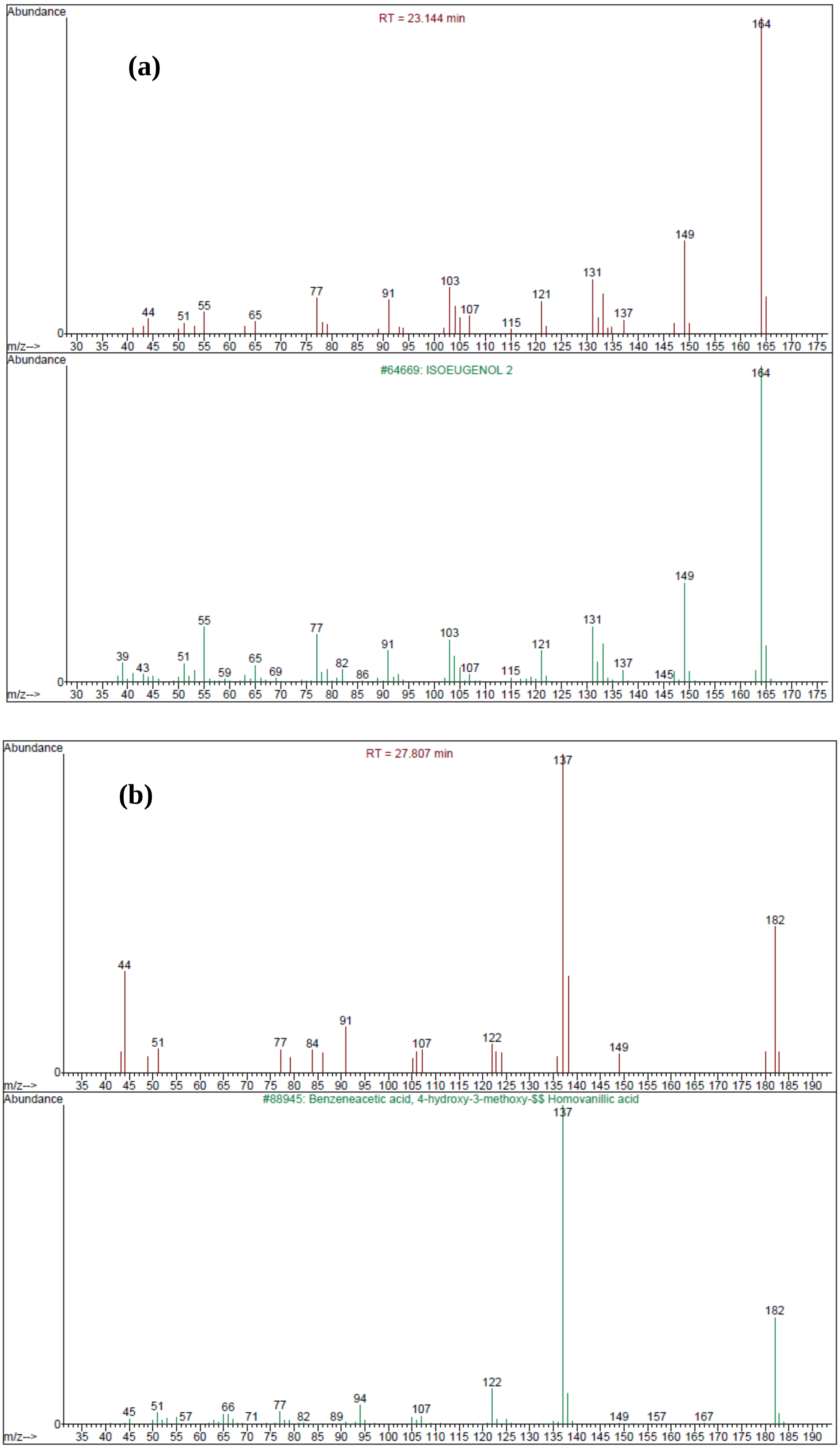

Fig. S7 (a) Mass spectra from the peak 23.14 and the standard isoeugenol. (b) Mass spectra from the peak 27.81 and the standard homovanillic acid.

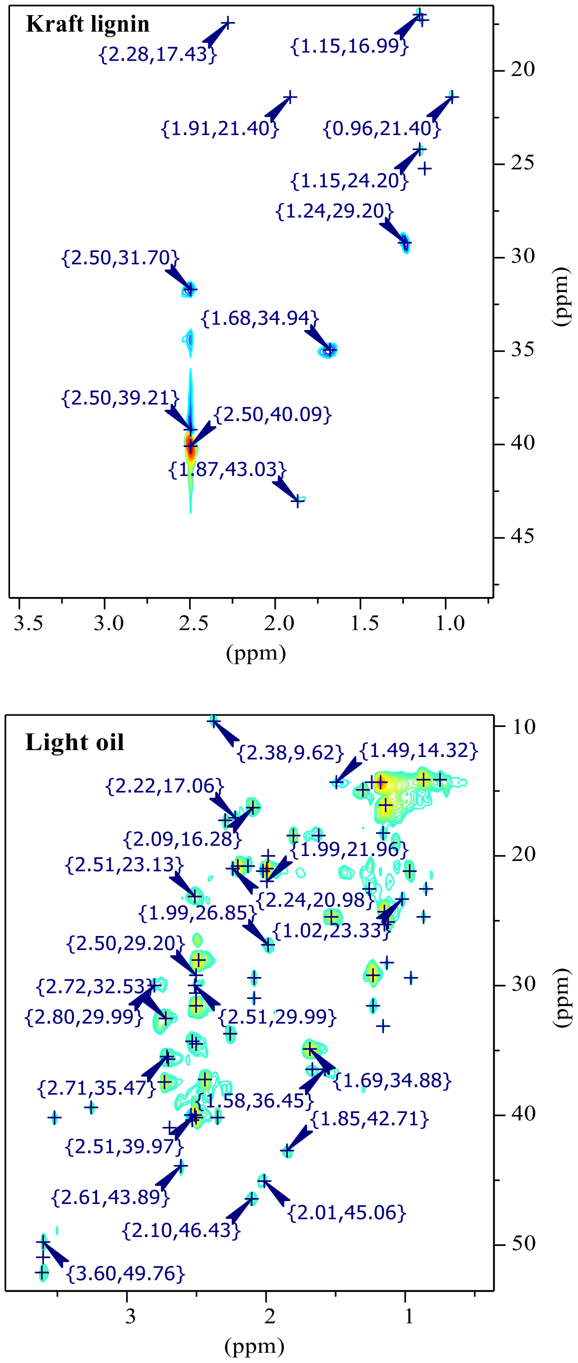

Fig. S8 Assignments of peaks/peak picking in the aliphatic hydrogenated regions of the Kraft lignin and light oil fraction obtained at 290 °C, 4h and CoMo/SBA-15 catalyst.

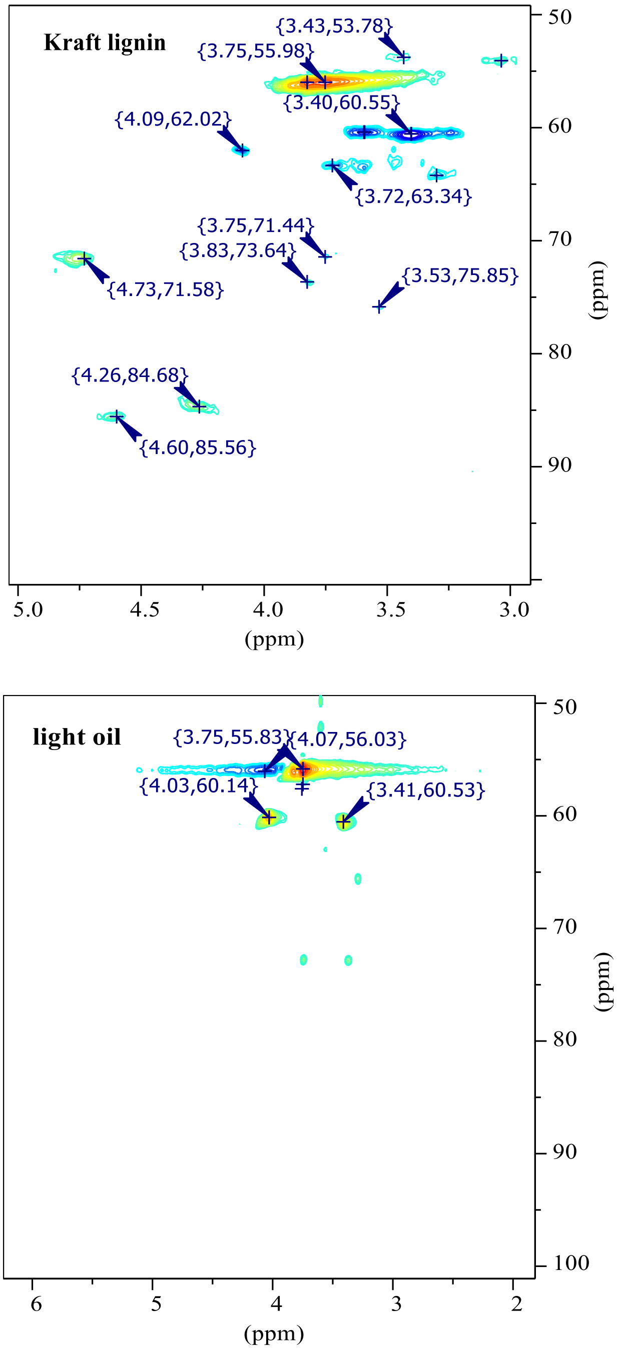

Fig. S9 Assignments of peaks/peak picking in the oxygenated and side-chain regions of the Kraft lignin and light oil obtained at 290 °C, 4h and CoMo/SBA-15 catalyst.

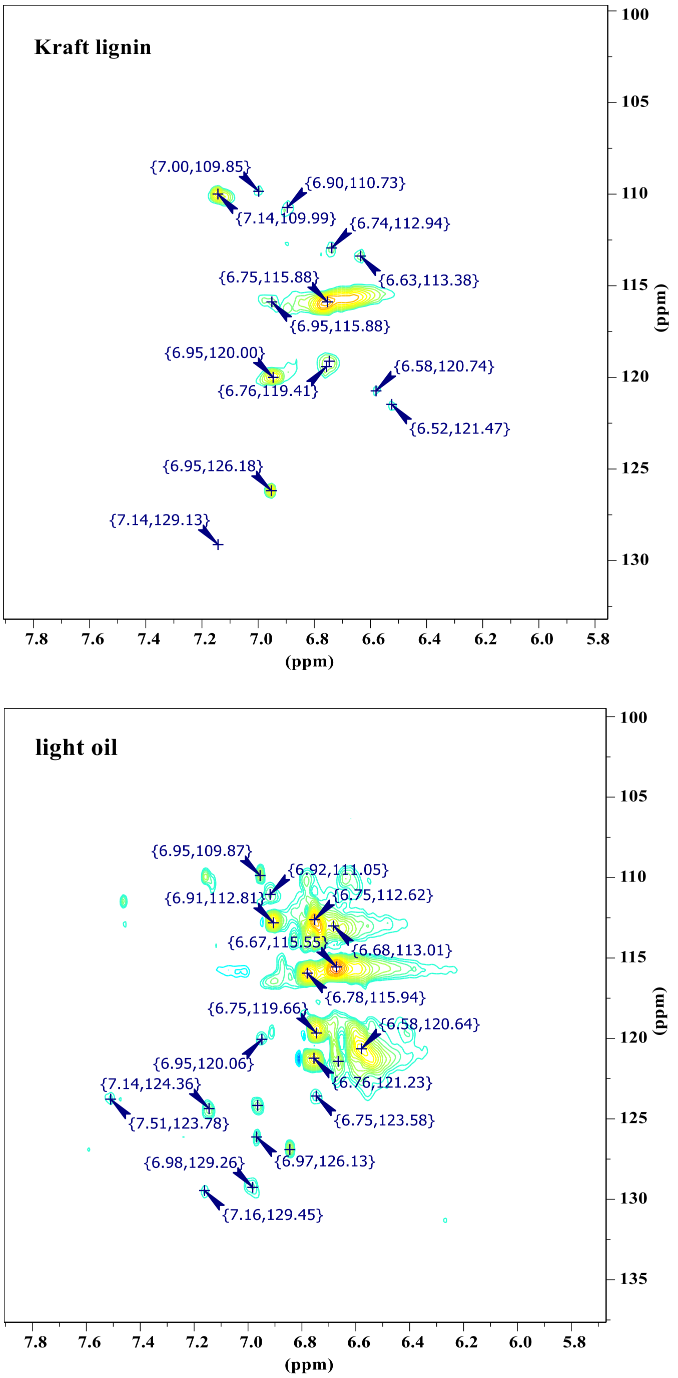

Fig. S10 Assignments of peaks/peak picking in the aromatic regions of the Kraft lignin and light oil obtained at 290 °C, 4h and CoMo/SBA-15 catalyst.

Table S1. The ICP−OES results of the prepared catalyst.

Catalyst	Metal content (wt%)	
	Mo	Co
SBA-15	-	-
Mo/SBA-15	9.8	-
Co/SBA-15	-	9.7
CoMo/SBA-15	4.9	4.8

Table S2. Results of KL depolymerization under different reaction conditions.

Parameters	Conditions	Heavy-oil (wt%)	Light-oil (wt%)	Solid residue (wt%)	Gas (wt%)
Time (h)	0.5	19.82 ± 0.25	10.26 ± 0.10	63.12 ± 1.03	6.33 ± 0.22
	2	22.75 ± 0.30	21.00 ± 0.27	48.50 ± 0.88	7.65 ± 0.16
	3	18.32 ± 0.19	27.00 ± 0.35	44.81 ± 0.73	10.02 ± 0.60
	4	17.88 ± 0.22	29.17 ± 0.50	41.50 ± 0.59	11.80 ± 0.17
	6	19.25 ± 0.18	25.39 ± 0.44	45.06 ± 0.37	10.61 ± 0.29
Temperature (°C)	240	17.60 ± 0.75	15.00 ± 0.26	61.02 ± 1.53	6.70 ± 0.32
	260	14.75 ± 0.24	21.00 ± 0.33	56.50 ± 1.15	8.11 ± 0.18
	270	13.17 ± 0.70	25.00 ± 0.28	52.81 ± 0.88	9.40 ± 0.56
	290	17.88 ± 0.13	29.17 ± 0.70	41.50 ± 0.57	11.40 ± 0.66
	320	19.70 ± 0.23	26.00 ± 0.64	44.06 ± 0.68	10.01 ± 0.49
Catalyst	Control	17.88 ± 0.51	29.17 ± 0.68	41.50 ± 0.67	11.00 ± 0.28
	SBA-15	15.60 ± 0.33	32.60 ± 0.56	38.50 ± 0.79	13.00 ± 0.14
	Co/SA-15	13.70 ± 0.43	38.90 ± 0.75	33.50 ± 0.38	14.20 ± 0.55
	Mo/SBA-15	12.96 ± 0.11	43.50 ± 0.61	31.70 ± 0.77	14.01 ± 0.39
	CoMo/SBA-15	8.42 ± 0.17	48.20 ± 0.82	28.50 ± 0.26	14.60 ± 0.24

Table S3. FT-IR spectra of KL and bio-oils obtained under different reaction conditions.

Wavenumber (cm^{-1})	Characteristics
3093–3619	O–H group.
2928	C–H (aliphatic and aromatic).
2831	C–H (methoxy group).
1712/1733	C=O in unconjugated ketones, carbonyl and ester groups).
1602	aromatic ring (C=C) vibration.
1512	aromatic ring (C=C) vibration.
1460	–OCH_3
1270, 1150, 1118	G ring and C–O vibrations.
1035	C–O vibration in ether, acid or ester groups.
860-741	para–substituted aromatic ring.

Table S4. Assignment of ^{13}C–^{1}H cross-signal in the HSQC spectra of Kraft lignin.

label	δ_C/δ_H	assignment
CH_3	0-1/10-16	CH_3 γ to Ar
CH_3, CH_2	1-1.4/16-23	CH_3, CH_2 β to Ar, CH γ to Ar
CH_2, CH	1.4-1.9/23-30	CH_2, CH β to Ar
CH_3, CH_2, CH	1.9-3.5/30-52.5	CH_3, CH_2, CH α to Ar
C_β	53.78/3.43	C_β–H_β in phenylcoumaran substructures (C)
B_β	54.07/3.04	C_β–H_β in resinol substructures (B)
–OCH_3	55.98/3.75	C– H in methoxyl
A_γ	60.55/3.40 and 60.40/3.59	C_γ–H_γ in β–O–4′ substructures (A)
D_γ	62.02/4.09	C_γ–H_γ in coniferyl alcohol substructures (D)
C_γ	63.34/3.72 and 64.23/3.30	C_γ–H_γ in phenylcoumaran substructures (C)
A_α	71.58/4.73	C_α–H_α in β–O–4′ substructures (A)
B_γ	71.44/3.75, 73.64/3.83 and 75.85/3.53	C_γ–H_γ in resinol substructures (B)
A_β	84.68/4.26	C_β–H_β in β–O–4′ substructures (A)
B_α	85.56/4.60	C_α–H_α in resinol substructures (B)
G_2'	109.99/7.14	C_2–H_2 in oxidized guaiacyl units (G′)
G_2	113.38-109.99/6.63-6.90	C_2–H_2 in guaiacyl units (G)
G_5	115.88/6.75	C_2–H_2 in guaiacyl units (G)
G_6	119.41-121.47/6.52-6.76	C_6–H_6 in guaiacyl units (G)
G_6'	120.00/6.95	C_6–H_6 in oxidized guaiacyl units (G′)
G_4	126.13/6.97	C_2–H_2 in guaiacyl units (G)
$H_{2,6}$	129.13/7.14	$C_{2,6}$–$H_{2,6}$ in *p*-hydroxyphenyl units (H)

Table S5. Elemental analyses of Kraft lignin and bio-oil fractions.

Entry	Sample	Elemental compositions (%)					HHV (MJ/kg)[d]
		C	H	O	N	S	
1	KL	61.69	6.09	30.09	0.65	1.48	25.92
2	Control (heavy oil)[a]	65.47	6.86	26.23	0.39	1.05	28.44
3	Mo/SBA-15 (heavy oil)[b]	67.61	6.92	24.81	0.32	0.34	29.24
4	CoMo/SBA-15 (heavy oil)[c]	67.74	7.29	24.44	0.41	0.12	29.75
5	CoMo/SBA-15 (light oil)[c]	71.23	8.31	20.16	0.23	0.07	32.60

[a] *Reaction conditions = 3.0g lignin, 90 mL ethanol, 290 ℃ and 4h.*

[b] *Reaction conditions = 3.0g lignin, 90 mL ethanol, 290 ℃, 4h and 0.3 g Mo/SBA-15 catalyst (10 wt%).*

[c] *Reaction conditions = 3.0g lignin, 90 mL ethanol, 290 ℃, 4h and 0.3g CoMo/SBA-15 catalyst (10 wt%).*

[d] *HHV (MJ/kg) = [(34 × C)+(124.3 × H)+(6.3 × N)+(19.3 × S)-(9.8 × O)]/100*
